# Quercetin and low-dose gamma irradiation modulate oxidative stress, inflammation, autophagy, and apoptosis in DEN-induced hepatocarcinogenesis

**DOI:** 10.1186/s12263-026-00810-2

**Published:** 2026-06-18

**Authors:** Nayera S. Khatab, Nermeen M. Elbakary, Mohamed Ali, Rokaya E. Maarouf, Faten Zahran

**Affiliations:** 1 Biochemistry Department, Faculty of Science, Zagazig University, Zagazig, 44519 Egypt; 2 Radiation Biology Department, National Center for Radiation Research and Technology (NCRRT), Egyptian Atomic Energy Authority, Cairo, Egypt

**Keywords:** Hepatocellular carcinoma, Diethylnitrosamine rat model, NF-κB p65, P38 MAPK, Caspase-3, ATG5, LC3II, Nutrigenomics, Gene expression

## Abstract

**Background:**

Diethylnitrosamine (DEN) induces hepatic injury and can promote hepatocarcinogenesis. Quercetin (QR) and low-dose gamma irradiation (LDR) have been explored as hepatoprotective and anticancer-modulating agents, but their combined effects in DEN-induced liver cancer have not been previously examined.

**Aim:**

To, for the first time, evaluate the novel combination of QR with LDR and compare its efficacy to QR or LDR alone in modulating DEN-induced liver cancer.

**Methods:**

Hepatic injury was induced in ninety male rats by intraperitoneal DEN administration (75 mg/kg weekly for 3 weeks, then 100 mg/kg weekly for 3 weeks). After 6 weeks, hepatic injury establishment was confirmed histopathologically in a subset of rats. Animals were then randomized into five groups for an 8-week treatment period: (C) normal control; (DEN) no further treatment; (DEN + QR) quercetin (50 mg/kg/day, oral); (DEN + IR) fractionated low-dose γ-irradiation (0.25 Gy once weekly for 4 weeks, total 1 Gy); (DEN + QR+IR) combined QR + IR. The total experimental duration was 14 weeks.

**Results:**

DEN induced severe hepatotoxicity, with significantly elevated serum ALT and AST compared to controls (*p* < 0.001). Markers of oxidative stress were markedly increased, while antioxidant enzymes and GSH were significantly reduced (*p* < 0.001). DEN also led to substantial activation of inflammatory and growth signaling pathways, including NF-κB, STAT-3, mTOR, and p38 MAPK (*p* < 0.001). Autophagy markers ULK-1, LC3-II, and ATG5 were significantly suppressed, and a pro-survival shift was evident with reduced caspase-3 activity and increased BCL-2 expression (*p* < 0.001).Treatment with quercetin (DEN + QR) or low-dose γ-irradiation (DEN + IR) alone significantly mitigated these alterations versus DEN, restoring liver enzymes, reducing oxidative stress and inflammation, and promoting apoptotic signaling (*p* < 0.001). The combination therapy (DEN + QR+IR) produced the most pronounced effects, with greater normalization of all biochemical and molecular parameters compared to DEN and to either monotherapy (*p* < 0.001). These changes were statistically superior to those observed with quercetin or irradiation alone (*p* < 0.01). Flow cytometry confirmed enhanced apoptosis and altered cell cycle dynamics in the combination group. Histopathological analysis showed markedly improved hepatic architecture and the lowest pathology scores with combination therapy (*p* < 0.001 vs. DEN).

**Conclusion:**

This study reveals, for the first time, a combined hepatoprotective effect of quercetin with low-dose gamma irradiation in DEN-induced liver pathology, suggesting new mechanistic insights and promising translational potential for combined therapy in DEN-associated liver injury and hepatocarcinogenesis.

**Clinical Trial:**

This work isn’t a clinical Trial.

**Graphical Abstract:**

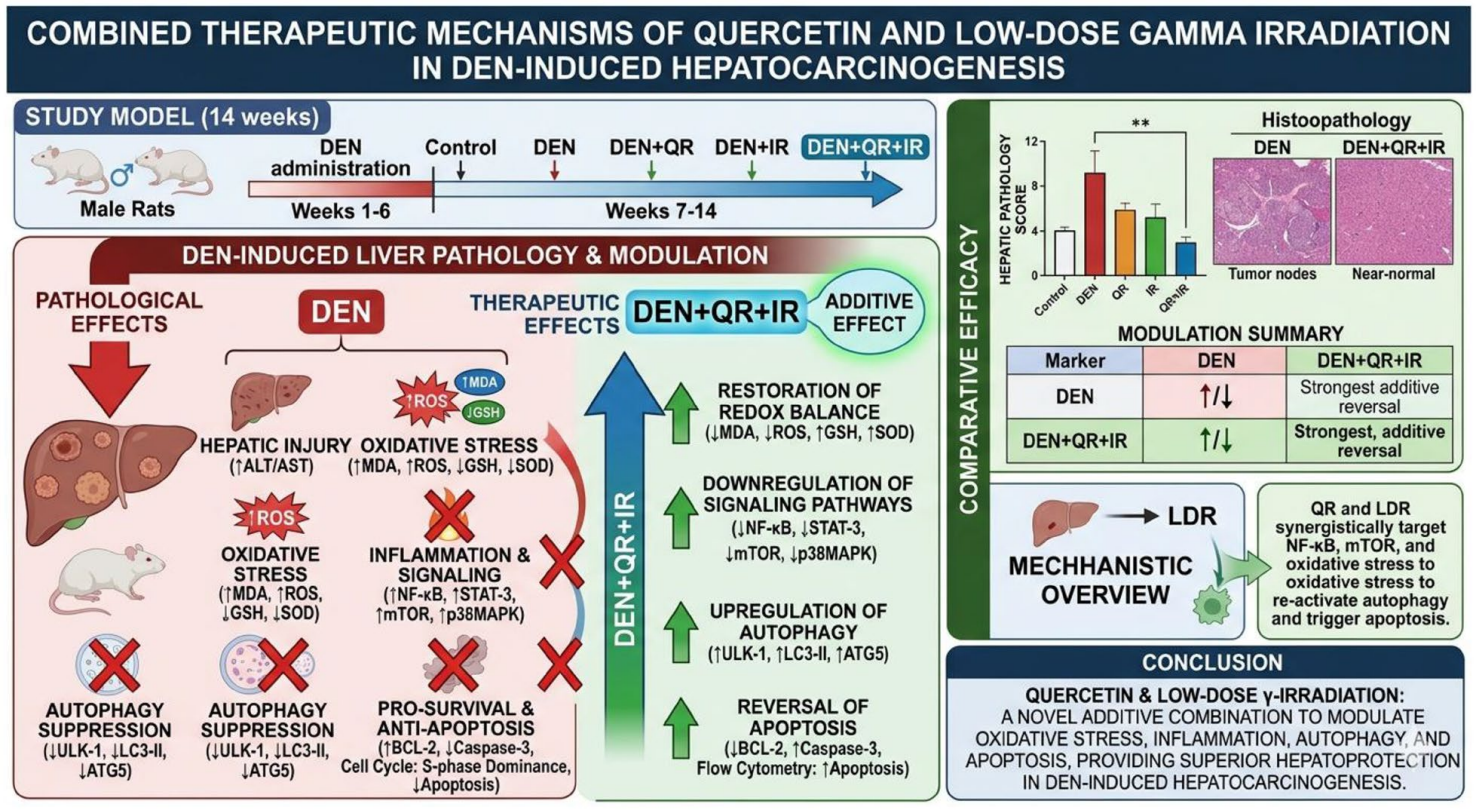

## Introduction

Hepatocellular carcinoma (HCC), the predominant primary liver malignancy, typically develops from chronic liver injury driven by genetic, epigenetic, and environmental factors [1]. Diethylnitrosamine (DEN) is a potent hepatocarcinogen widely used to model this process in rodents, where it induces oxidative stress, inflammation, autophagy dysregulation, and apoptosis inhibition that collectively promote hepatocarcinogenesis [2–4].

Conventional HCC therapies face well-documented clinical limitations. First, radiotherapy is constrained by the high radiosensitivity of normal liver parenchyma, with a whole-liver tolerance dose (TD5/5) of only 30 Gy, which is below the dose required for tumor control in most cases [5]. Second, sorafenib, the first-line systemic therapy, shows primary or acquired resistance in over 60% of patients, mediated by compensatory activation of EGFR/IGF-1R signaling and pro-survival autophagy [6]. Third, curative options such as surgical resection and radiofrequency ablation are limited by 5-year recurrence rates exceeding 70% due to underlying cirrhosis and multicentric hepatocarcinogenesis [7]. These limitations underscore the need for novel strategies with improved therapeutic indices. Recent studies have highlighted the hepatoprotective potential of dietary phytochemicals and medicinal plants through antioxidant and anti-inflammatory mechanisms [8–11]. Quercetin, a naturally occurring flavonoid, exhibits potent hepatoprotective effects in liver carcinogenesis. In DEN-induced rat models, quercetin attenuates hepatic inflammation by suppressing NF-κB activation and reduces oxidative DNA damage [12]. It modulates the tumor microenvironment and inhibits HCC growth by targeting NF-κB and mTOR pathways [13]. In HCC cells, it inhibits proliferation by blocking the mTOR/PI3K/Akt pathway and induces autophagic cell death [14], and sensitizes HepG2 cells to sorafenib by downregulating pro-survival autophagy [15]. However, its efficacy in DEN-induced hepatocarcinogenesis, particularly in combination with radiation-based modalities, remains unexplored.

Low-dose gamma irradiation (LDR) elicits adaptive responses, enhancing DNA repair and immune function without cytotoxicity [16]. While low-dose gamma radiation (LDR) has been shown to attenuate DEN-induced hepatic inflammation and fibrosis via NF-κB/TNF-α/ MAPK inhibition [17], its impact on autophagy and apoptosis in DEN-induced hepatocarcinogenesis remains uncharacterized. Separately, quercetin exerts chemopreventive effects in DEN models, but has never been evaluated in combination with radiation in any oncological context. Consequently, a significant knowledge gap exists: it is unknown whether LDR can enhance quercetin’s bioactivity against HCC, or whether quercetin can augment the hormetic effects of LDR.

To address this gap, the present study tested the hypothesis that combined quercetin (QR) and fractionated LDR would provide superior hepatoprotection against DEN-induced liver cancer compared to either monotherapy. To our knowledge, this represents the first investigation of a flavonoid + LDR combination in liver cancer. We assessed hepatic function (ALT, AST), oxidative stress (SOD, GSH, MDA, ROS), inflammatory signaling (NF-κB, STAT-3, mTOR, P38 MAPK), autophagy (ULK-1, LC3-II, ATG5), apoptosis (caspase-3, BCL-2), cell cycle dynamics (flow cytometry), and histopathology to elucidate potential interactive mechanisms.

## Materials and methods

### Materials

Quercetin (Sigma-Aldrich; ≥95% purity, Product Number: Q4951; CAS Number: 117-39-5) was obtained from Sigma-Aldrich, United States. All remaining analytical-grade chemicals and reagents used in the study were also sourced from Sigma-Aldrich (St. Louis, MO, USA).

### Radiation facility and irradiation protocol

The experimental rats underwent whole-body gamma irradiation totaling 1 Gy, delivered in four fractions of 0.25 Gy each, weekly over four consecutive weeks after tumor establishment. Irradiation was performed at the National Center for Radiation Research and Technology (NCRRT) using a Canadian Gamma-Cell-40 (Cs-137) biological irradiator (Canada Ltd., Ottawa, Ontario). The dose rate was calibrated to 0.403 Gy/min following the Protection and Dosimetry Department guidelines.

The dose of 0.25 Gy per fraction was selected as it falls within the low-dose radiation range (< 1.0 Gy acute exposure) as defined by the United Nations Scientific Committee on the Effects of Atomic Radiation [UNSCEAR], and within the hormetic window (0.01–0.5 Gy) previously shown to induce adaptive antioxidant and anti-inflammatory responses in rodent liver without causing overt cytotoxicity or DNA damage [18, 19]. Fractionation was employed to allow for cellular recovery and amplification of adaptive responses between doses, while the cumulative 1.0 Gy total dose remains well below the threshold for radiation-induced hepatic fibrosis (> 8 Gy) [20]. This protocol has been shown to activate antioxidant pathways and suppress NF-κB signaling in DEN-induced hepatocarcinogenesis models [21].

### Animals

Male adult Wistar albino rats (weight 120–130 g) were sourced from the NCRRT breeding unit. Animals were acclimatized and maintained on a standard commercial pellet diet with ad libitum access to water for one week prior to experimentation.

### Ethics approval statement

All animal care and use complied with the National Institutes of Health guidelines (NIH No. 85:23, revised 1996) and the Ethics Committee of the National Center for Radiation Research and Technology (NCRRT), Atomic Energy Authority, Cairo, Egypt. The study adhered to CIOMS, ICLAS (2012), and ARRIVE guidelines 2.0, and followed the 3Rs principles (replacement, reduction, refinement) for animal experimentation. The approved protocol serial number is F/7/EC/25, approved on 19/03/2025.

### DEN-Induced hepatocarcinogenesis

Hepatocellular carcinoma (HCC) was induced by intraperitoneal injection of diethyl nitrosamine (DEN) at 75 mg/kg body weight once weekly for 3 weeks, followed by 100 mg/kg body weight once weekly for an additional 3 weeks, as described by Sherif et al. [22].

At the end of week 6, prior to any therapeutic intervention, one rat was randomly selected from the DEN-induction cohort and euthanized to confirm model establishment. Liver sections were stained with H&E to verify the presence of preneoplastic foci and dysplastic hepatocytes, and serum ALT/AST were measured. Only after histological confirmation were the remaining 79 DEN-exposed rats randomized to treatment groups. This confirmatory animal was excluded from all subsequent analyses. The healthy control group was not sampled at week 6 to avoid unnecessary euthanasia, consistent with 3Rs principles.

### Experimental design and randomization

Ninety male albino rats (120–130 g) were used. Animals were randomly allocated using a computer-generated sequence (GraphPad QuickCalcs) into two initial cohorts: (1) Healthy Control group (*n* = 10) receiving isotonic saline i.p. and (2) DEN-induction cohort (*n* = 80) receiving DEN to induce hepatocarcinogenesis.

After 6 weeks and histological confirmation of hepatocarcinogenesis from one randomly selected DEN rat, the remaining 79 DEN-exposed rats were randomly re-allocated using a second computer-generated sequence into four therapeutic groups (*n* = 20, 20, 20, 19) to receive different treatments for 8 weeks. This two-stage randomization ensures that all treatment groups had equivalent DEN exposure at baseline. To confirm successful randomization, baseline body weights were compared across groups and showed no significant differences (one-way ANOVA, *p* = 0.84).

**Scheme 1:**
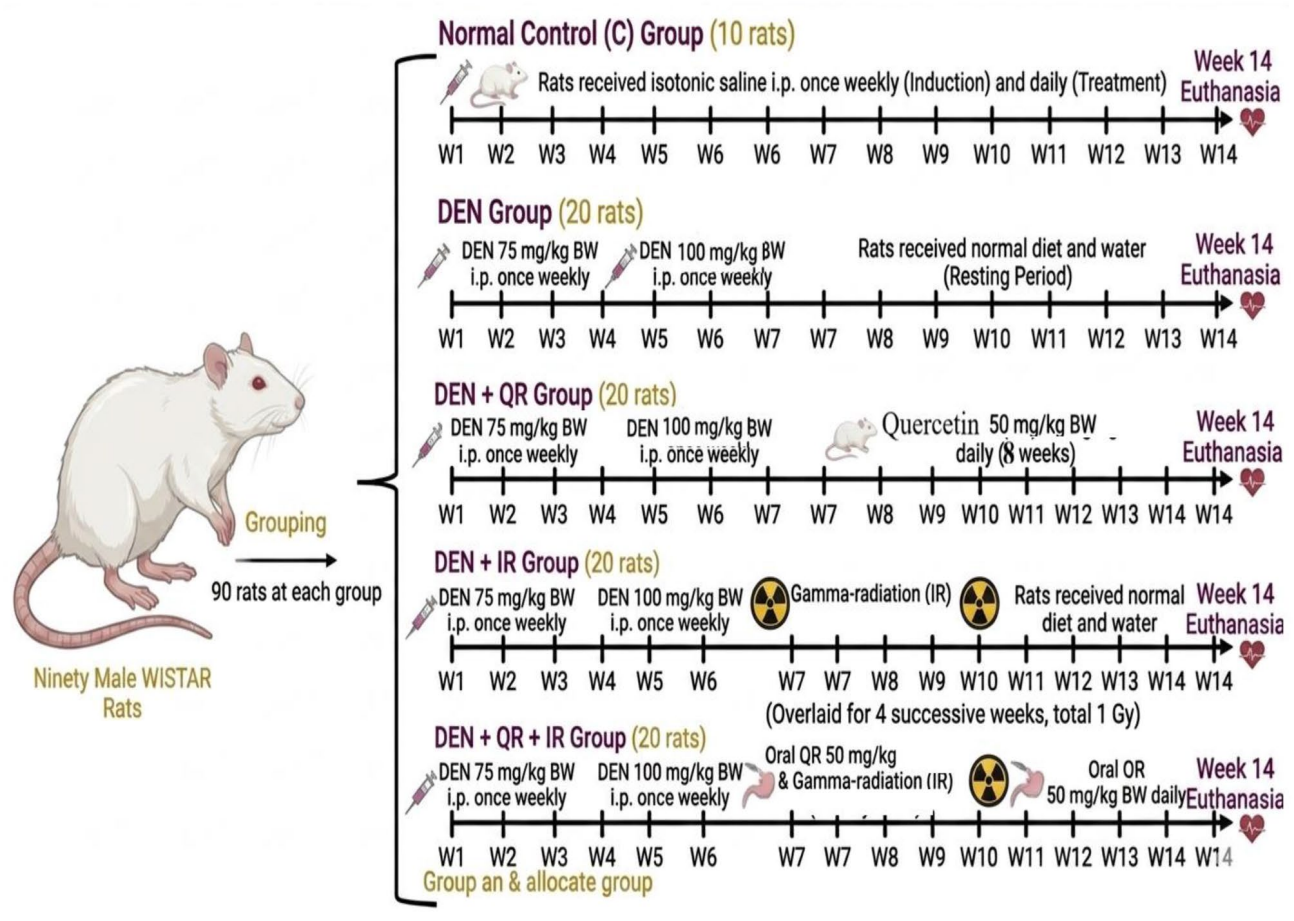
Schematic diagram of the experimental design and timeline

Group assignments were:

- Group 1 (C): Normal control (10 rats), receiving i.p isotonic saline.
- Group 2 (DEN): DEN exposure (75 mg/kg body weight once weekly for 3 weeks, followed by 100 mg/kg body weight once weekly for an additional 3 weeks).
- Group 3 (DEN + QR): DEN exposure followed by daily oral quercetin at 50 mg/kg for 8 weeks, starting one week after liver cancer induction (Omar et al. 2025) (20 rats).
- Group 4 (DEN + IR): DEN exposure followed by exposure to fractionated low-dose gamma radiation (0.25 Gy) four times over four successive weeks, totaling 1 Gy (20 rats).
- Group 5 (DEN + QR + IR): DEN exposure with both gamma irradiation and quercetin treatment as described (20 rats) (Scheme 1).

Initial group sizes were *n* = 10 for healthy controls and *n* = 20 for all DEN-treated groups. The *n* = 20 for DEN groups was based on expected 15–40% mortality from chronic DEN toxicity and replicates the design of our previous DEN-HCC study approved by the same Ethics Committee [23]. The study protocol was approved by the Ethics Committee of NCRRT, Atomic Energy Authority, Cairo, Egypt (Serial #F/7/EC/25, 19/03/2025) and adhered to NIH, CIOMS, ICLAS, and ARRIVE 2.0 guidelines. Final survivors at study termination were: Control *n* = 10, DEN *n* = 6, DEN + QR *n* = 14, DEN + LDR *n* = 16, DEN + QR+LDR *n* = 17.

### Serum and tissue collection, and preparation of crude tissue homogenates

At the end of the 14-week experimental period, rats were anesthetized with urethane (1.2 g/kg body weight). Blood was collected via cardiac puncture into anticoagulant tubes, and then centrifuged at 1500 × g for 10 min at 4 °C (Hettich Universal 32 A, Germany). Serum was stored at -20 °C for subsequent analyses. Liver tissues were dissected, washed with normal saline, weighed, and homogenized in ice-cold phosphate buffer (0.1 M, pH 7.4) at a 1:10 (w/v) ratio using a Teflon homogenizer. Cytoplasmic and nuclear proteins were extracted using the NE-PER kit (Thermo Scientific, Cat. #78833). Total protein content was determined by the Lowry method [24]. Additionally, representative liver tissue sections from each group were fixed in 10% formaldehyde for histopathological analysis.

Histopathological evaluation was performed in a blinded manner. All tissue slides (H&E) were coded by an independent researcher who was not involved in data collection or analysis. A board-certified pathologist then evaluated and scored all specimens without knowledge of group allocation or treatment status to eliminate assessment bias. The codes were broken only after completion of all scoring.

Biochemical parameters were measured using a fully automated clinical chemistry analyzer according to manufacturer protocols to minimize operator-dependent bias. Flow cytometry data were acquired and analyzed using predefined gating strategies, applied uniformly across all samples by an analyst blinded to group identity.

### Assessment of liver function

Serum levels of alanine aminotransferase (ALT) and aspartate aminotransferase (AST) were measured using colorimetric assays based on Reitman and Frankel [25]. Commercial kits were used (Biodiagnostic, Egypt; ALT: Cat # AL 1031; AST: Cat # AS 1061). Briefly, serum samples were reacted with 2,4-dinitrophenylhydrazine (1 mmol/L) and incubated at 37 °C for 30 min. Absorbance was read at 505 nm with a double-beam spectrophotometer (Thermo Electron UV-Visible, England).

### Biochemical assays

Hepatic oxidative stress Malondialdehyde (MDA) and antioxidant biomarkers including Superoxide Dismutase (SOD), Reduced Glutathione (GSH), were determined in the prepared liver tissue homogenate supernatant .

Oxidative status was assessed by measuring malondialdehyde (MDA) via the thiobarbituric acid reactive substances (TBARS) assay at 532 nm, following Yoshioka et al. [26].

Total superoxide dismutase (SOD) activity was determined per Sun et al. [27] based on inhibition of the reduction of Nitro Blue Tetrazolium (NBT) by superoxide generated in the xanthine–xanthine oxidase system. SOD activity is expressed as units per milligram of protein (U/mg).

Glutathione (GSH) levels in liver were quantified using rat ELISA kits (MyBioSource, San Diego, USA) GSH: Cat. # MBS265966 Protocols followed the manufacturers’ guidelines.

### Reactive oxygen species (ROS)

Hepatic oxidative stress status was evaluated by measuring levels of reactive oxygen species-derived oxidative products in liver homogenates using a commercial ELISA kit (MBS2802061, MyBioSource, San Diego, USA) according to the manufacturer’s instructions. This assay detects total Reactive Oxygen Species (ROS) (U/mg protein) as an indirect index of oxidative stress, utilizing a quantitative sandwich mechanism. The dynamic linear range of the assay is 0.625 U/mL – 40 U/mL with a minimum analytical sensitivity threshold of 0.31 U/mL. The final ROS concentrations were normalized against the total protein content of the tissue homogenate and expressed as U/mg protein.”

### Quantification of inflammatory cytokines

Hepatic levels of total NF-κB p65 (MyBioSource, Cat# MBS287521, USA) and total STAT3 (MyBioSource, Cat# MBS760293, San Diego, USA) were quantified using commercial rat-specific ELISA kits according to the manufacturer’s instructions. Liver homogenates were prepared as described previously, and total protein concentration was determined. Results were normalized to total protein content and expressed as ng/mg protein.

### Autophagy markers

- Liver levels of ULK-1 was measured using rat-specific ELISA kits (MyBioSource, San Diego, USA):
  - ULK-1: Cat. # MBS7279343 Protocols followed the manufacturers’ guidelines.
- LC3-II (The lapidated form) levels were quantified using a selective ELISA kit (Cat. #CBA-5116, Cell Biolabs, San Diego, CA) which utilizes a specialized lysis and wash protocol to remove cytosolic LC3-I, ensuring the specific measurement of the lipidated, autophagosome-associated LC3-II form.”

### Quantitative real-time PCR (qRT-PCR)

Total RNA was extracted from liver tissue homogenates using the RNeasy Plus Mini Kit (Qiagen, Venlo, Netherlands) following the manufacturer’s instructions. Reverse transcription was performed with the First Strand cDNA Synthesis Kit (Thermo Scientific, USA). qRT-PCR was carried out using RNA-direct SYBR Green Master Mix (Invitrogen™) on an Mx3000P qPCR system (Agilent Technologies, California, USA). Primers targeting murine mTOR, P38 MAPK, and ATG5 are listed in Table 1. Cycle threshold (Ct) values were normalized to the housekeeping gene GAPDH. Relative gene expression levels were calculated using the 2 − ΔΔCT method [28].

**Table 1:** Primers used for qRT-PCR

Primer	Sense (5′-3′)	Antisense (5′-3′)	Accession number
mTOR	CTGCACTTGTTGTTGCCTCC	ATCTCCCTGGCTGCTCCTTA	NM_019906.2
P38 MAPK	TCGGCACACTGATGACGAAA	TCATGGCTTGGCATCCTGTT	NM_031020.3
ATG5	GGATGGGACTGCAGAATGATTT	GTGTCATGCTTCGGTGTCCT	NM_001014250.2﻿
GAPDH	GCGAGATCCCGCTAACATCA	ATTCGAGAGAAGGGAGGGCT	NM_017008.4

### Cell cycle and Annexin V detection of apoptosis by flow cytometry

#### Flow cytometry preparation

- Liver tissue was dissected and minced, then enzymatically dissociated with 0.5 mg/mL collagenase IV (Sigma) in PBS for 25 min at 37 °C on a shaker.
- The suspension was centrifuged at 500 × g for 10 min; the supernatant was discarded, and the pellet was washed twice with PBS.
- The cells were filtered through a 100 μm cell strainer to obtain a single-cell suspension, washed with cell staining buffer, and centrifuged at 400 × g for 5 min at 4 °C.
- The pellet was resuspended in cell staining buffer, and cell count/viability were assessed using trypan blue with a bright-line hemocytometer, adjusting to 1 × 10^6 cells/mL.
- Cells were harvested, washed twice in ice-cold PBS, and fixed overnight in 70% ethanol at 4 °C.
- After fixation, cells were washed in PBS, collected by centrifugation, and stained with propidium iodide (PI; 50 µg/mL) for cell cycle analysis.

For cell cycle analysis, cells were fixed overnight and stained with propidium iodide to quantify DNA content.

For apoptosis detection, fresh cells were stained with the Annexin V-FITC/PI Apoptosis Detection Kit (BD Biosciences) to distinguish live, early apoptotic, late apoptotic, and necrotic populations.

### Annexin V Apoptosis Detection

The “FITC Annexin V Apoptosis Detection Kit with PI” (BD Pharmingen™, BD Biosciences, USA; Catalog #51-66121E) was used for flow cytometry apoptosis analysis in accordance with the manufacturer’s instructions. In addition to cell morphology, apoptosis can also be detected by flow cytometric examination of heterogeneous cell populations that retain cell viability. This technique can differentiate intact, apoptotic, or dead cells resulting from either necrosis or apoptosis all at once. Annexin V is a calcium-dependent phospholipid-binding protein with a strong affinity for phosphatidylserine (PS), which is normally found on the inner side of the plasma membrane. Translocation of PS from the inner to the outer layer of the plasma membrane, exposing it at the external cell surface, is one of the difficult-to-detect alterations that characterize early apoptotic stages. In addition to apoptosis, necrosis also exhibits this tendency. The distinction between these two forms of cell death lies in the fact that, while the cell membrane remains intact during the early stages of apoptosis, it breaks down during necrosis, allowing propidium iodide (PI) to enter, which can be used to identify necrotic cells.

In summary, Cells were resuspended in 1× Binding Buffer at 1 × 10^6 cells/mL. Following that, 10 µL of 20 mg/mL propidium iodide (PI) and 5 µL of FITC Annexin V were added to flow cytometry tubes, and the tubes were incubated for 15 min at room temperature in the dark. The number of apoptotic cells was measured using a flow cytometer (BD Accuri C6), and the data were subsequently analyzed using CellQuest software on a FACSC-LSR (Becton & Dickinson Company).

### Histopathological analysis

For histopathological evaluation, liver specimens were fixed in 10% neutral buffered formalin, processed routinely, and embedded in paraffin. Sections (4–5 μm thick) were stained with hematoxylin and eosin (H&E) [29]. A board-certified veterinary pathologist, blinded to the treatment groups, evaluated the slides using a Leica DM500 microscope with Leica Application Suite software. Histopathological scoring followed a modified scheme based on Lobenhofer et al. [30], assessing five parameters: hepatocyte necrosis, fibrosis, cellular infiltration, hepatocyte apoptosis, and hepatocyte fatty change. A single composite total score from 0 to 3 was assigned to each section based on overall severity, where 0 = normal/absent, 1 = mild, 2 = moderate, and 3 = marked/severe changes. For each group, liver sections from six different animals were randomly selected for semi-quantitative assessment. Statistical analysis of total pathology scores was performed using the Kruskal-Wallis test followed by Dunn’s multiple comparisons post-hoc test, with *p* < 0.05 considered statistically significant.

### Statistical analysis

- Results are presented as mean ± SEM. Differences among groups were analyzed by one-way ANOVA followed by Tukey-Kramer post hoc tests. Analyses were performed with GraphPad Prism version 8 (La Jolla, CA). Significance was set at *p* < 0.05. Data were analyzed for histopathology scores (*n* = 6 rats/group) using the Kruskal-Wallis test followed by Dunn’s multiple comparisons post-hoc test.
- For biochemical and molecular assays, to maintain a balanced statistical design and avoid selection bias from unequal survivor numbers, *n* = 6 samples per group were analyzed. These samples were randomly selected from all surviving animals in each group using a second computer-generated randomization list (Research Randomizer v4.0). This sample size provided > 80% power to detect the observed effect sizes for primary endpoints, as confirmed by post-hoc power analysis (G*Power 3.1.9.7).

## Results

### Effects of quercetin and low-dose γ-irradiation on DEN-induced hepatic dysfunction

**Fig. 1:**
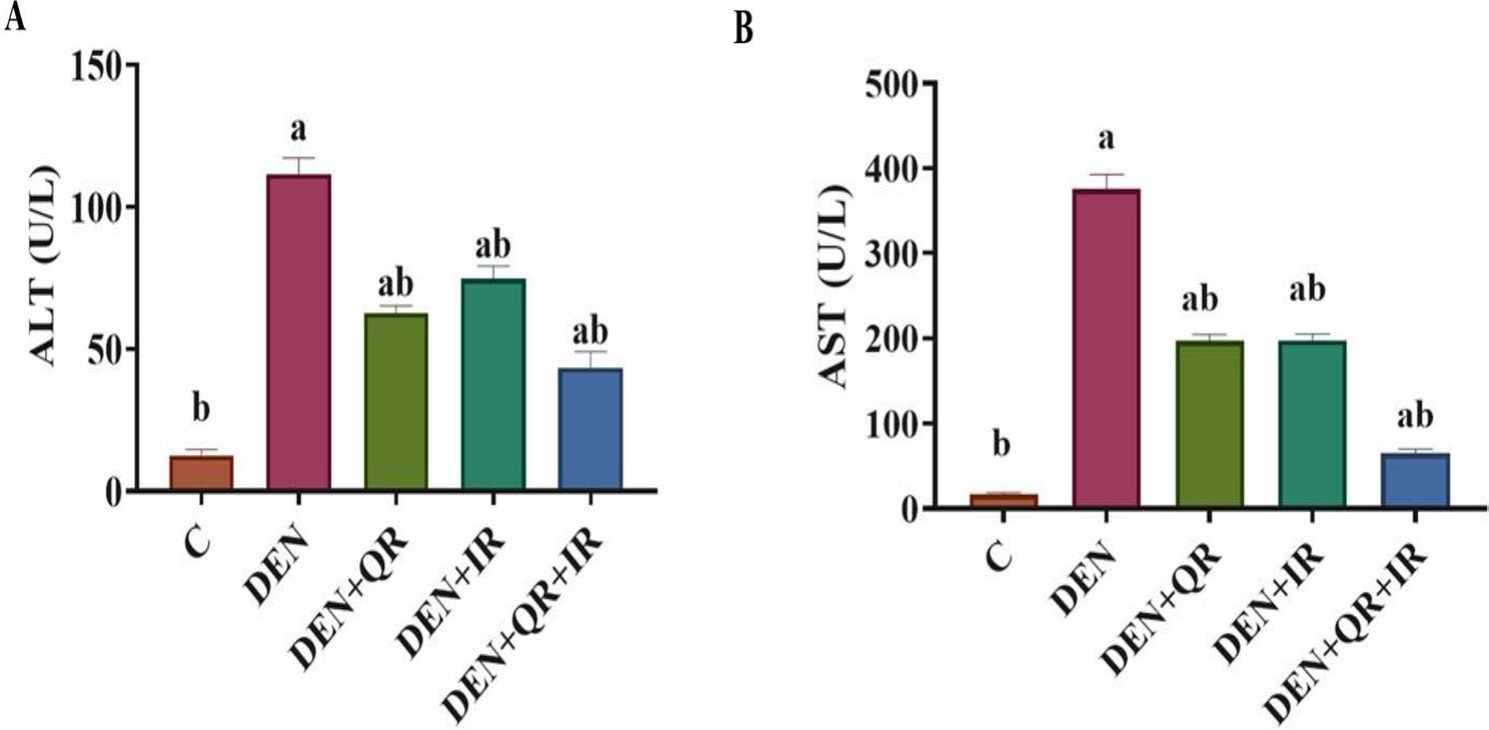
Effect of quercetin and low dose gamma irradiation on liver enzymes ALT, AST. Control, DEN: diethylnitrosamine injected rat, DEN + QR: Diethylnitrosamine injected rat and treated with Quercetin, DEN + IR: Diethylnitrosamine injected rat and exposed to low dose gamma irradiation, DEN + QR+IR: Diethylnitrosamine injected rat treated with quercetin and exposed to low dose gamma irradiation, Values are the mean ± SEM (*n* = 6). a:Statistically significant compared to the Normal Control (C) group. b:Statistically significant compared to the DEN-induced group

Serum alanine aminotransferase (ALT) and aspartate aminotransferase (AST) were assessed as markers of hepatocellular injury (Fig. 1A and B). DEN administration resulted in a 9.1-fold increase in ALT and a 22.4-fold increase in AST compared to Control (*p* < 0.001 for both), confirming severe hepatocyte damage. Treatment with quercetin (DEN + QR) reduced ALT by 43.9% and AST by 47.6% versus DEN alone (*p* < 0.001 for both). Despite this reduction, ALT remained 4.9-fold and AST remained 11.8-fold higher than Control levels (*p* < 0.001 for both). Low-dose γ-irradiation (DEN + IR) decreased ALT by 33.1% and AST by 47.4% compared to DEN (*p* < 0.001 for both). However, ALT was 5.9-fold and AST was 11.8-fold higher than Control (*p* < 0.001 for both).The combination therapy (DEN + QR+IR) produced the greatest amelioration of liver enzyme elevation, reducing ALT by 61.2% and AST by 82.7% relative to DEN (*p* < 0.001 for both). This reduction was significantly greater than that achieved by either DEN + QR (*p* < 0.01 for ALT; *p* < 0.001 for AST) or DEN + IR (*p* < 0.001 for both). In the DEN + QR+IR group, ALT was 3.4-fold and AST was 3.9-fold higher than Control (*p* < 0.001 for both). These findings indicate that combination therapy produced greater hepatoprotective effects than either monotherapy (Fig. 1A, B).

### QR and LDR ameliorate DEN-Induced disruption of hepatic redox homeostasis

**Fig. 2:**
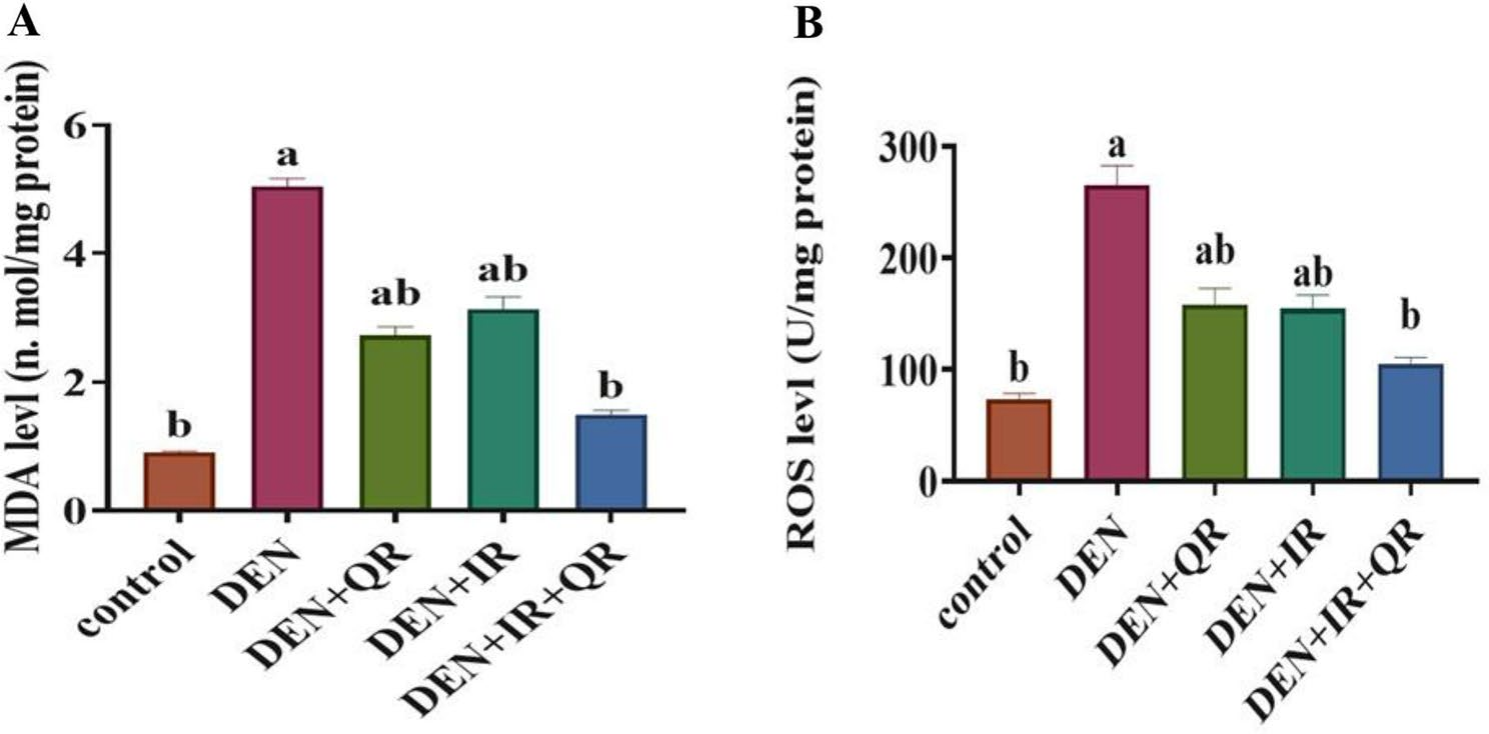
Effect of Quercetin and low dose gamma irradiation on oxidative stress. control, DEN: Diethylnitrosamine injected rat, DEN + QR: Diethylnitrosamine injected rat and treated with Quercetin, DEN + IR: Diethylnitrosamine injected rat and exposed to low dose gamma irradiation, DEN + QR+IR: Diethylnitrosamine injected rat treated with quercetin and exposed to low dose gamma irradiation, Values are the mean ± SEM (*n* = 6). a: Statistically significant compared to the Normal Control (C) group. b: Statistically significant compared to the DEN-induced group

**Fig. 3:**
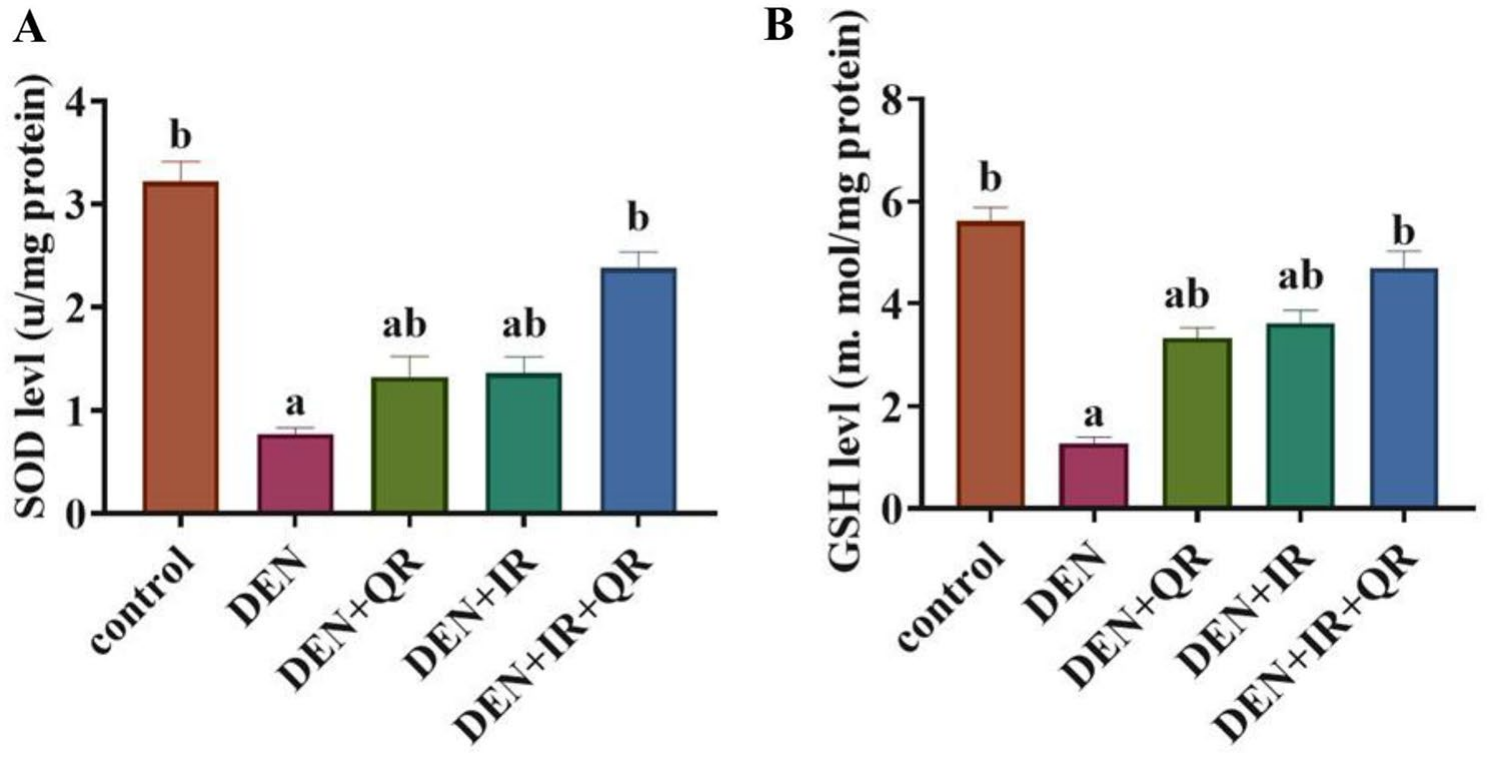
Effect of Quercetin and low dose gamma irradiation on antioxidant level. control, DEN: Diethylnitrosamine injected rat, DEN + QR: Diethylnitrosamine injected rat and treated with Quercetin, DEN + IR: Diethylnitrosamine injected rat and exposed to low dose gamma irradiation, DEN + QR+IR: Diethylnitrosamine injected rat treated with quercetin and exposed to low dose gamma irradiation, Values are the mean ± SEM (*n* = 6). a: Statistically significant compared to the Normal Control (C) group. b: Statistically significant compared to the DEN-induced group

Hepatic oxidative stress and antioxidant status were evaluated by measuring malondialdehyde (MDA), reactive oxygen species (ROS), superoxide dismutase (SOD) activity, and reduced glutathione (GSH) content (Figs. 2A-B and 3A-B).

DEN exposure induced a 5.6-fold increase in MDA and a 3.6-fold increase in ROS compared to Control (*p* < 0.001 for both), indicating extensive lipid peroxidation and oxidative stress. Quercetin co-administration (DEN + QR) reduced MDA by 46.0% and ROS by 40.3% versus DEN alone (*p* < 0.001 for both). However, MDA remained 3.0-fold and ROS remained 2.1-fold higher than Control (*p* < 0.001 for both). Low-dose γ-irradiation (DEN + IR) decreased MDA by 38.1% and ROS by 41.8% compared to DEN (*p* < 0.001 for both). MDA was 3.5-fold and ROS was 2.1-fold higher than Control (*p* < 0.001 for both).The combination therapy (DEN + QR+IR) produced the greatest attenuation of oxidative damage, reducing MDA by 70.2% and ROS by 60.5% relative to DEN (*p* < 0.001 for both). This reduction was significantly greater than that achieved by DEN + QR (*p* < 0.001 for both) or DEN + IR (*p* < 0.001 for both). In the DEN + QR+IR group, MDA was 1.7-fold and ROS was 1.4-fold higher than Control (*p* < 0.01 for both) (Fig. 2A, B).

On the other hand, SOD and GSH Conversely, DEN depleted hepatic SOD activity by 76.1% and GSH content by 77.2% versus Control (*p* < 0.001 for both). QR monotherapy restored SOD by 74.0% and GSH by 160.2%, while IR monotherapy restored SOD by 76.6% and GSH by 182.0% relative to DEN (*p* < 0.001 for all).The combination (DEN + QR+IR) achieved the largest recovery of antioxidant defenses, increasing SOD by 209.1% and GSH by 267.2% versus DEN (*p* < 0.001 for both), which was significantly greater than either monotherapy (*p* < 0.01 for SOD; *p* < 0.001 for GSH) (Fig. 3A, B).

Combination therapy with quercetin and low-dose irradiation (DEN + QR+IR) resulted in significant normalization of redox homeostasis. Compared to the DEN group, DEN + QR+IR showed decreased MDA and ROS levels and increased GSH and SOD activities (*p* < 0.01 for all), approaching control values. The effects of combination therapy on these markers were greater than those observed with QR or IR monotherapy.

### Quercetin and low-dose γ-Irradiation attenuate DEN-induced inflammatory signaling via suppression of NF-κB and STAT-3 pathways

**Fig. 4:**
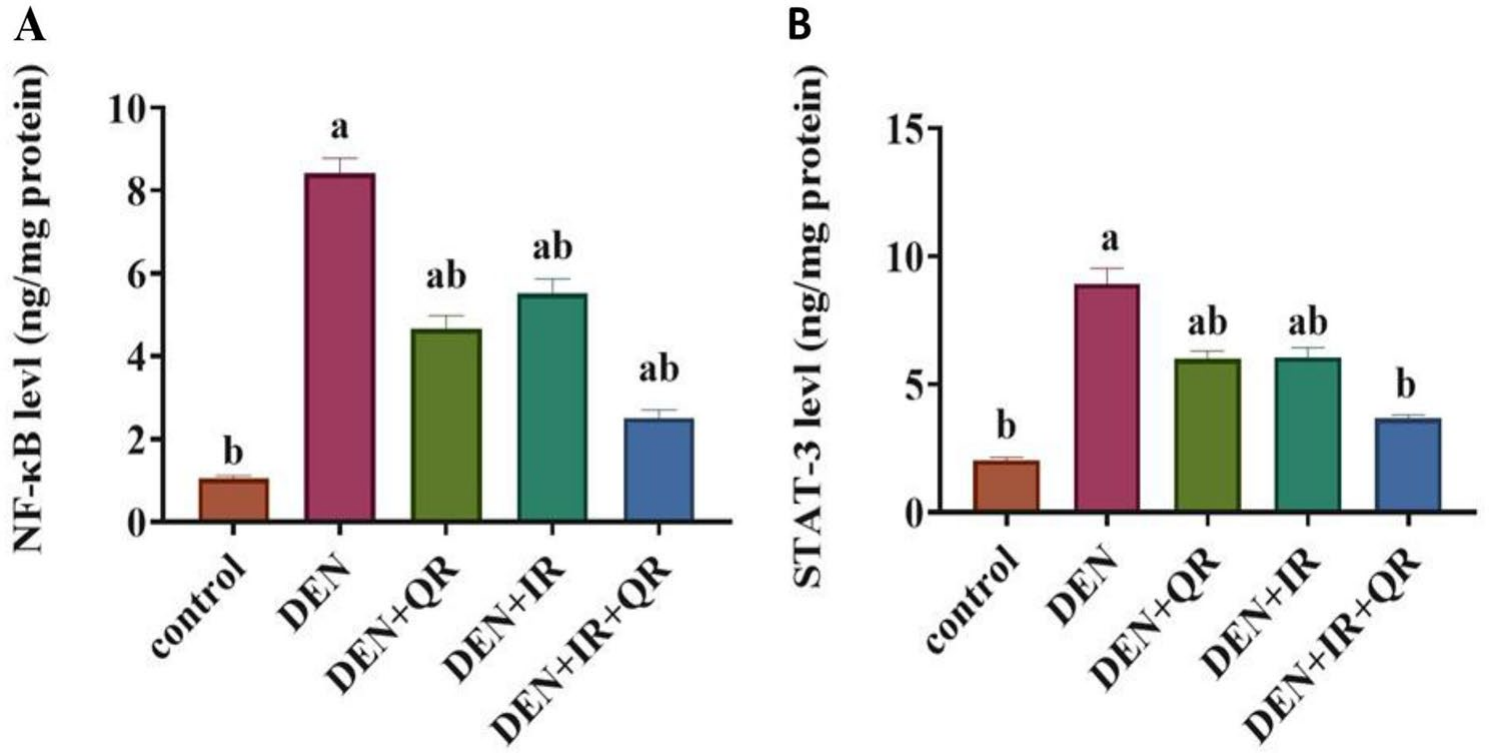
Effect of quercetin and low-dose gamma irradiation on hepatic total NF-κB p65 and STAT3 protein levels in DEN-induced rats. Control, DEN: Diethylnitrosamine injected rat, DEN + QR: Diethylnitrosamine injected rat and treated with Quercetin, DEN + IR: Diethylnitrosamine injected rat and exposed to low dose gamma irradiation, DEN + QR+IR: Diethylnitrosamine injected rat treated with quercetin and exposed to low dose gamma irradiation, Values are the mean ± SEM (*n* = 6). a: Statistically significant compared to the Normal Control (C) group. b: Statistically significant compared to the DEN-induced group

Hepatic NF-κB and STAT-3 activities were assessed as markers of inflammatory and pro-survival signaling (Fig. 4A and B).

DEN exposure induced an 8.0-fold increase in NF-κB and a 4.4-fold increase in STAT-3 activity compared to Control (*p* < 0.001 for both), consistent with activation of pro-inflammatory and pro-survival pathways. Quercetin administration (DEN + QR) reduced NF-κB by 44.8% and STAT-3 by 33.1% versus DEN alone (*p* < 0.001 for both). However, NF-κB remained 4.4-fold and STAT-3 remained 2.9-fold higher than Control levels (*p* < 0.001 for both). Low-dose γ-irradiation (DEN + IR) decreased NF-κB by 34.6% and STAT-3 by 32.4% compared to DEN (*p* < 0.001 for both). NF-κB was 5.2-fold and STAT-3 was 3.0-fold higher than Control (*p* < 0.001 for both). The combination therapy (DEN + QR+IR) produced the greatest suppression of inflammatory signaling, reducing NF-κB by 70.2% and STAT-3 by 59.0% relative to DEN (*p* < 0.001 for both). This reduction was significantly greater than that achieved by either DEN + QR (*p* < 0.001 for both) or DEN + IR (*p* < 0.001 for both). In the DEN + QR+IR group, NF-κB was 2.4-fold and STAT-3 was 1.8-fold higher than Control (*p* < 0.01 for both), indicating signaling was approaching but not fully normalized to baseline levels.

### Impact of quercetin and/or gamma irradiation on mTOR and P38 MAPK gene expression in DEN-Induced hepatic carcinogenesis

**Fig. 5:**
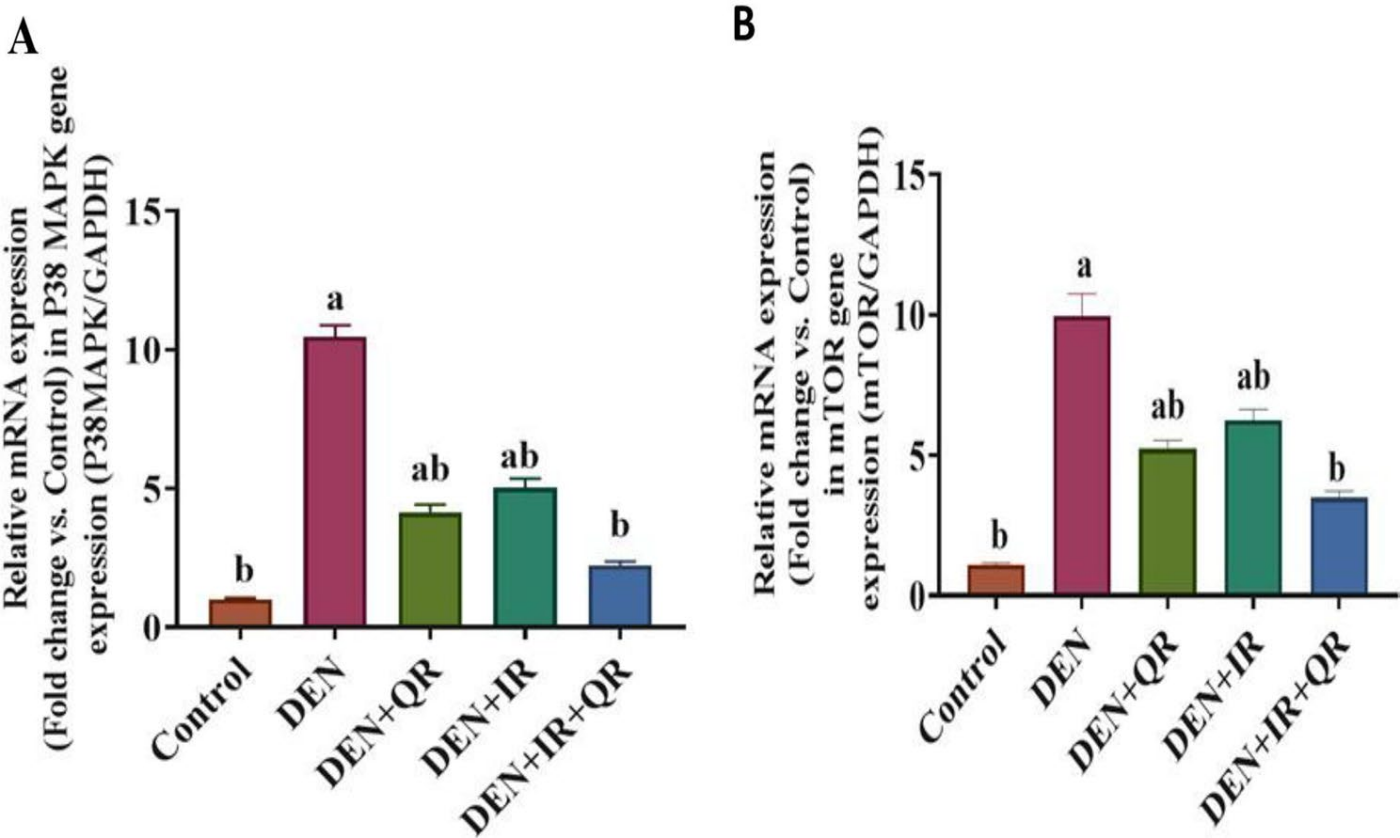
Effect of quercetin and low-dose gamma irradiation on hepatic p38MAPK and mTOR gene expression in DEN-induced rats. Relative mRNA expression levels of (A) p38 MAPK and (B) mTOR were determined by qRT-PCR and normalized to GAPDH as a reference gene. Data are expressed as fold change relative to the healthy Control group, which was set to 1.0 (*n* = 6 rats/group) control, DEN: Diethylnitrosamine injected rat, DEN + QR: Diethylnitrosamine injected rat and treated with Quercetin, DEN + IR: Diethylnitrosamine injected rat and exposed to low dose gamma irradiation, DEN + QR+IR: Diethylnitrosamine injected rat treated with quercetin and exposed to low dose gamma irradiation, Values are the mean ± SEM (*n* = 6). a: Statistically significant compared to the Normal Control (C) group. b: Statistically significant compared to the DEN-induced group

Hepatic mTOR and P38 MAPK transcript levels were quantified to assess pro-tumorigenic growth signaling and stress-activated kinase pathways (Fig. 5A and B).

DEN exposure induced a 10.6-fold increase in P38 MAPK and a 9.0-fold increase in mTOR transcript levels compared to Control (*p* < 0.001 for both), consistent with activation of proliferative and stress-response signaling. Quercetin administration (DEN + QR) reduced P38 MAPK by 60.5% and mTOR by 47.4% versus DEN alone (*p* < 0.001 for both). However, P38 MAPK remained 4.2-fold and mTOR remained 4.7-fold higher than Control levels (*p* < 0.001 for both). Low-dose γ-irradiation (DEN + IR) decreased P38 MAPK by 52.0% and mTOR by 37.3% compared to DEN (*p* < 0.001 for both). P38 MAPK was 5.1-fold and mTOR was 5.6-fold higher than Control (*p* < 0.001 for both).The combination therapy (DEN + QR+IR) produced the greatest suppression of growth signaling, reducing P38 MAPK by 78.6% and mTOR by 64.9% relative to DEN (*p* < 0.001 for both). This reduction was significantly greater than that achieved by either DEN + QR (*p* < 0.01 for P38 MAPK; *p* < 0.001 for mTOR) or DEN + IR (*p* < 0.001 for both). 

### QR and LDR restore DEN-suppressed hepatic autophagy

**Fig. 6:**
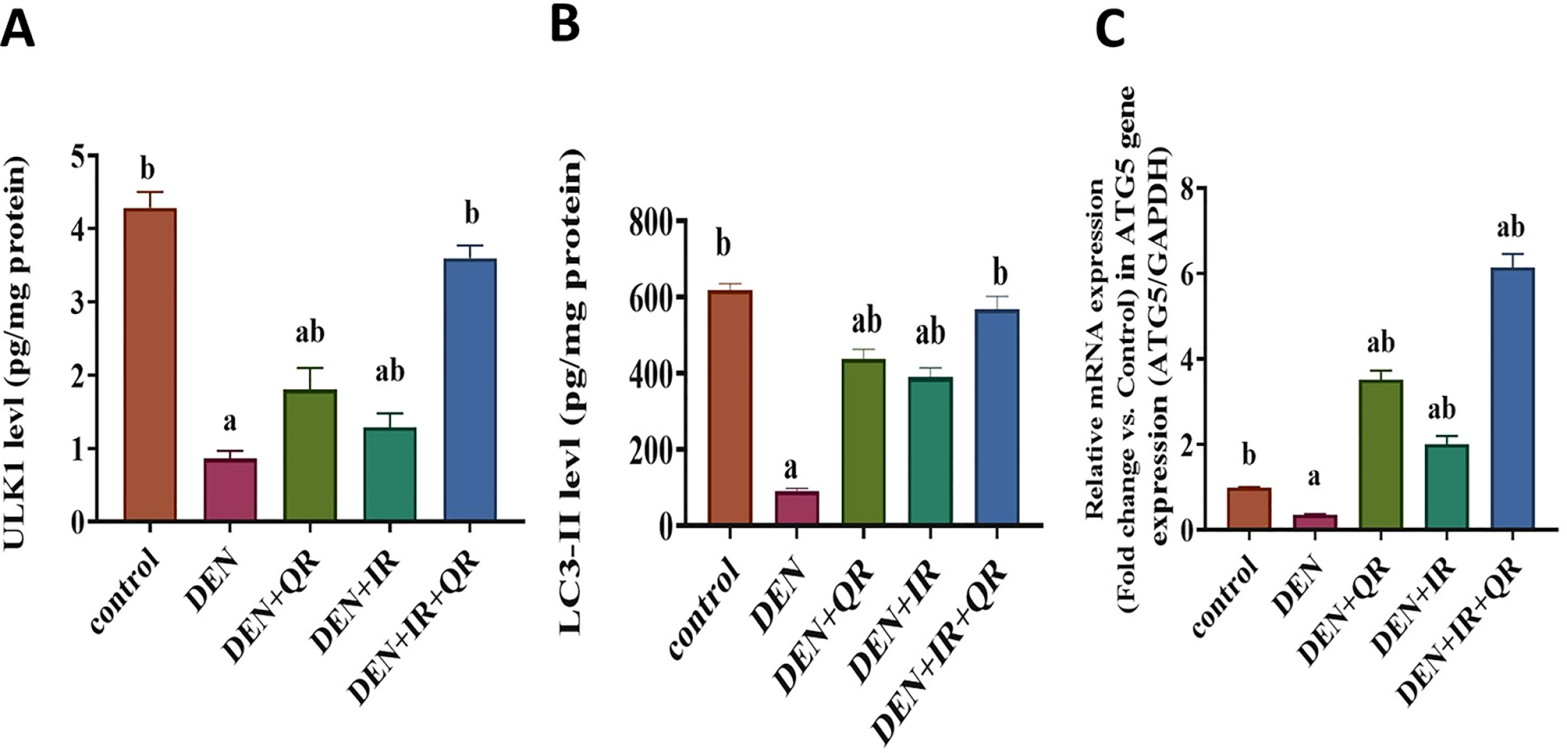
Effect of Quercetin and low dose gamma irradiation on ULK1, LC3-II levels and ATG5 gene expressions. Control, DEN: Diethylnitrosamine injected rat, DEN + QR: Diethylnitrosamine injected rat and treated with Quercetin, DEN + IR: Diethylnitrosamine injected rat and exposed to low dose gamma irradiation, DEN + QR+IR: Diethylnitrosamine injected rat treated with quercetin and exposed to low dose gamma irradiation, Values are the mean ± SEM (*n* = 6). a: Statistically significant compared to the Normal Control (C) group. b: Statistically significant compared to the DEN-induced group

Hepatic autophagy was evaluated by measuring ULK-1 protein level, LC3-II protein level, and ATG5 gene expression (Fig. 6A-C). DEN treatment suppressed autophagy markers, reducing ULK-1 by 80.0%, LC3-II by 85.5%, and ATG5 by 64.6% compared to Control (*p* < 0.001 for all), indicating impaired autophagy. Quercetin administration (DEN + QR) increased ULK-1 by 109.3%, LC3-II by 387.9%, and ATG5 by 902.9% versus DEN alone (*p* < 0.001 for all). Despite this increase, ULK-1 remained 58.0% lower and LC3-II remained 29.1% lower than Control (*p* < 0.001 for both), while ATG5 was 3.5-fold higher than Control (*p* < 0.001). Low-dose γ-irradiation (DEN + IR) elevated ULK-1 by 50.0%, LC3-II by 333.0%, and ATG5 by 471.4% compared to DEN (*p* < 0.001 for all). ULK-1 was 69.9% lower, LC3-II was 37.1% lower, and ATG5 was 2.0-fold higher than Control (*p* < 0.001 for all). The combination therapy (DEN + QR+IR) produced the greatest restoration of autophagy, increasing ULK-1 by 318.6%, LC3-II by 531.1%, and ATG5 by 1654.3% relative to DEN (*p* < 0.001 for all). This increase was significantly greater than that achieved by DEN + QR (*p* < 0.01 or ULK-1 and LC3-II; *p* < 0.001 for ATG5) or DEN + IR (*p* < 0.001 for all).

### QR and LDR Shift the balance toward pro-apoptotic signaling in DEN-treated liver

**Fig. 7:**
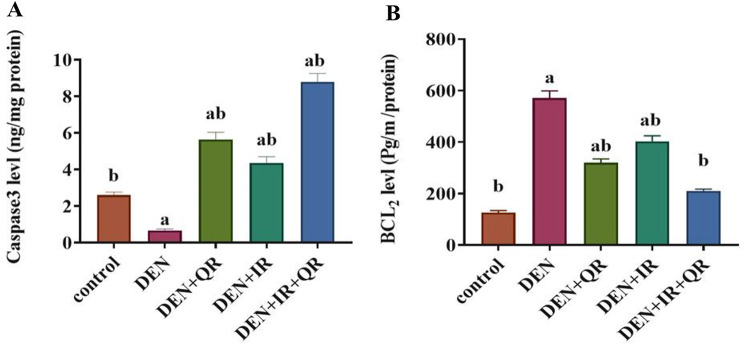
Effect of Quercetin and low dose gamma irradiation on Caspase-3, BCL-2 levels. control, DEN: Diethylnitrosamine injected rat, DEN + QR: Diethylnitrosamine injected rat and treated with Quercetin, DEN + IR: Diethylnitrosamine injected rat and exposed to low dose gamma irradiation, DEN + QR+IR: Diethylnitrosamine injected rat treated with quercetin and exposed to low dose gamma irradiation, Values are the mean ± SEM (*n* = 6). a: Statistically significant compared to the Normal Control (C) group. b: Statistically significant compared to the DEN-induced group

Hepatic apoptosis was evaluated by measuring caspase-3 activity and BCL-2 protein levels (Fig. 7A and B). DEN exposure suppressed pro-apoptotic signaling, reducing caspase-3 activity by 74.6% and increasing anti-apoptotic BCL-2 by 4.5-fold compared to Control (*p* < 0.001 for both), indicating a shift toward cell survival. Quercetin administration (DEN + QR) increased caspase-3 by 754.5% and reduced BCL-2 by 44.0% versus DEN alone (*p* < 0.001 for both). Caspase-3 was 2.2-fold higher and BCL-2 remained 2.5-fold higher than Control (*p* < 0.001 for both). Low-dose γ-irradiation (DEN + IR) elevated caspase-3 by 559.1% and decreased BCL-2 by 29.4% compared to DEN (*p* < 0.001 for both). Caspase-3 was 1.7-fold higher and BCL-2 was 3.2-fold higher than Control (*p* < 0.001 for both).The combination therapy (DEN + QR+IR) produced the greatest induction of pro-apoptotic signaling, increasing caspase-3 by 1231.8% and reducing BCL-2 by 63.2% relative to DEN (*p* < 0.001 for both). This effect was significantly greater than that achieved by DEN + QR (*p* < 0.01 for caspase-3; *p* < 0.001 for BCL-2) or DEN + IR (*p* < 0.001 for both).

### Quercetin potentiates low-dose gamma irradiation by modulating cell cycle dynamics and inducing apoptosis

**Fig. 8:**
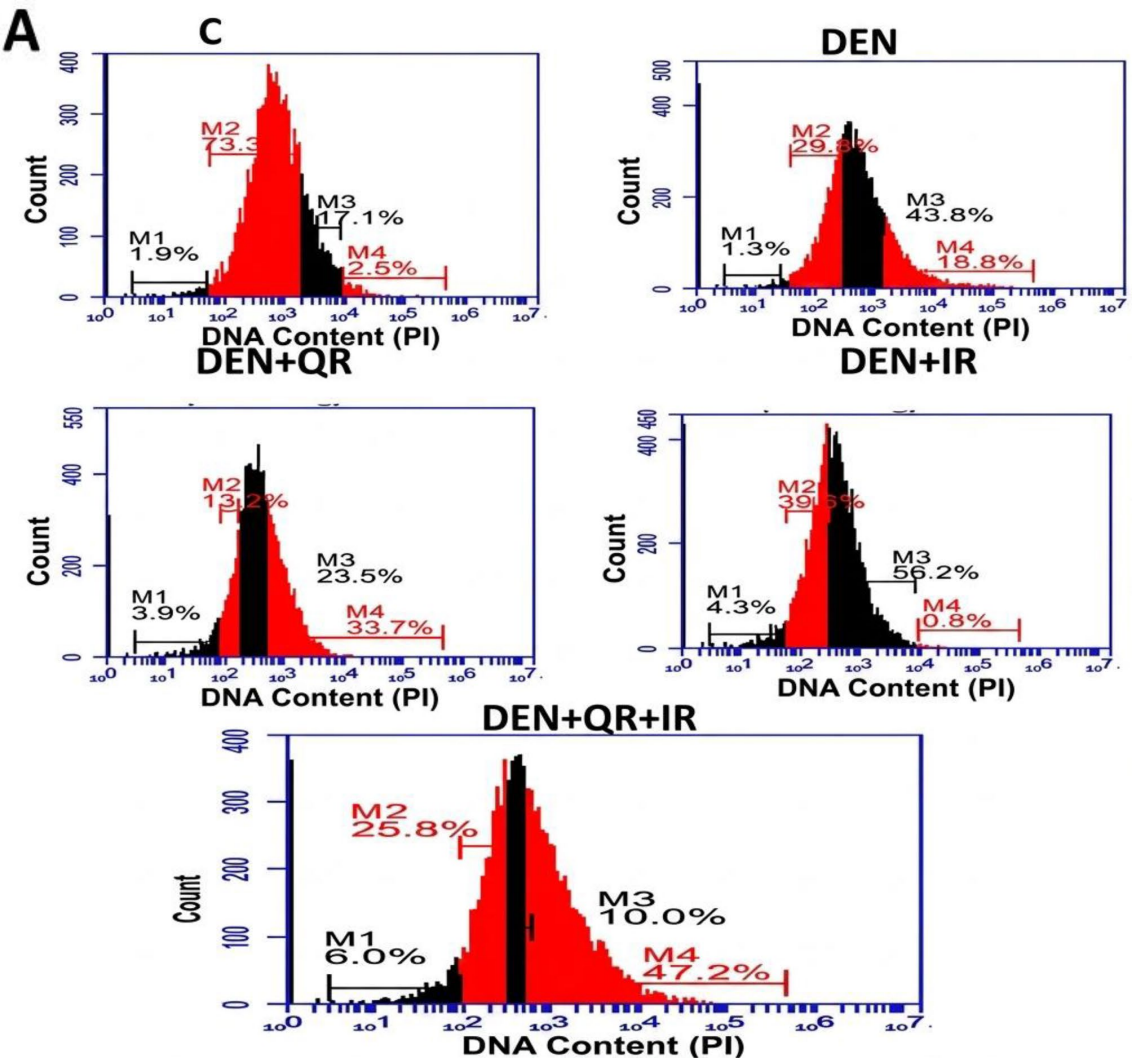

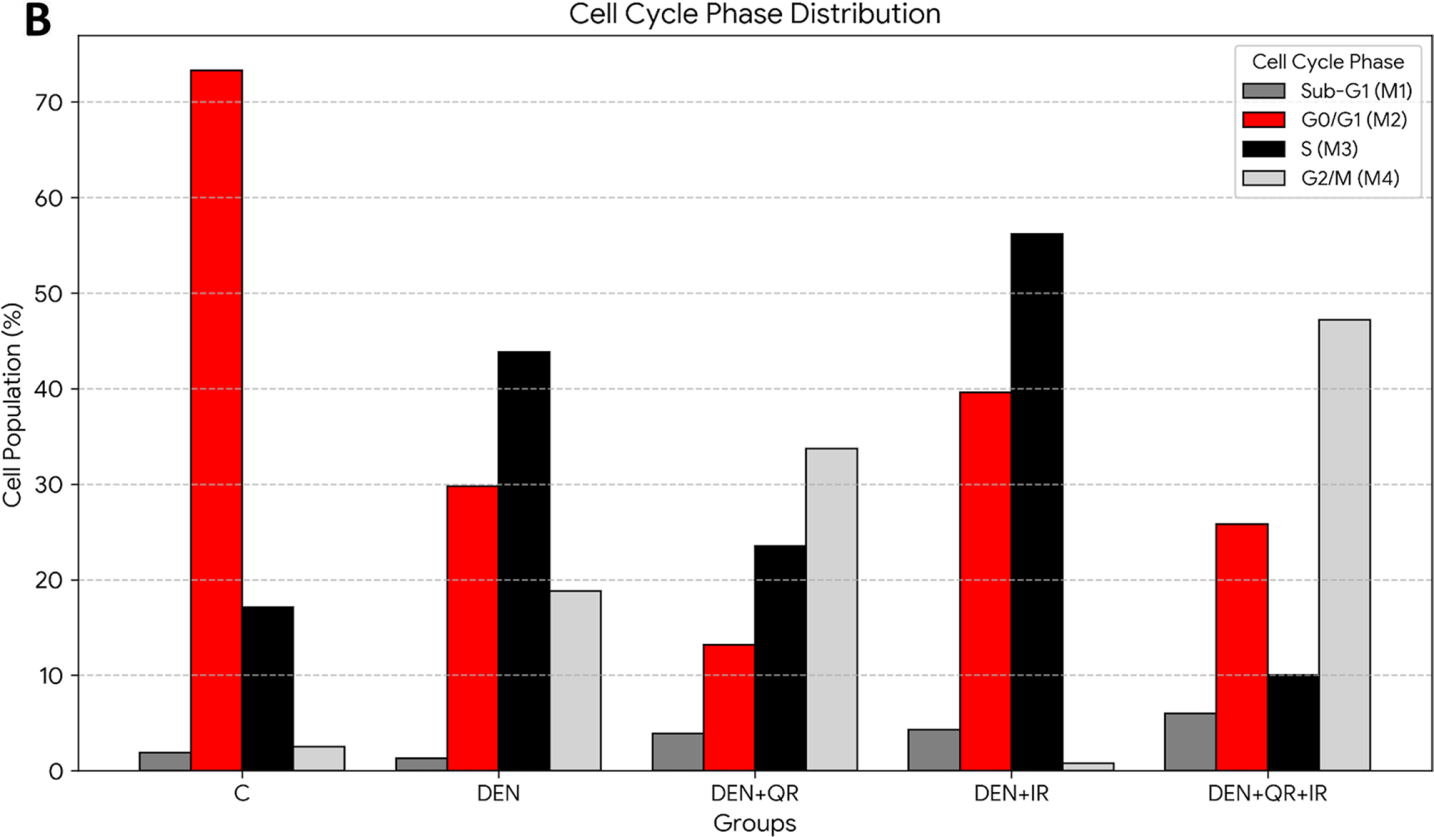
Quercetin and/or low dose gamma irradiation induced cell cycle arrest and apoptosis in liver cancer cells. (**A**) Representative flow cytometry histograms of cells treated with C (Control), DEN, DEN + QR, DEN + IR, and DEN + QR+IR. The x-axis has been updated to DNA Content (PI) as per the measurement of propidium iodide fluorescence, and the y-axis represents the cell Count. (**B**) Quantitative analysis of cell cycle distribution. The bar graph illustrates the percentage of cells in the G1/G0, S, and G2/M phases for each experimental group. Data are presented as the mean percentage of the total cell population. Figure 8 presents the cell cycle distribution including sub-G1 from PI staining of fixed cells

To elucidate the mechanisms underlying the anticancer efficacy of Quercetin (QR) and its role in sensitizing liver cancer cells to low-dose gamma irradiation (IR), we analyzed cell cycle distribution using flow cytometry. Given that the cell cycle is a fundamental regulator of tumor growth, metastasis, and recurrence, modulating phase-specific checkpoints and associated regulatory proteins remains a primary target in hepatocellular carcinoma (HCC) therapy. Representative histograms and quantitative phase distributions (Sub-G1, G0/G1, S, and G2/M) are illustrated in Fig. 8A and B.

### The impact of DEN and monotherapies on cell cycle progression

In the DEN-induced HCC group, flow cytometric analysis revealed a marked shift in cell cycle dynamics. Compared to the Control (C) group, which showed a normal distribution dominated by the G0/G1 phase (73.3%), the DEN group exhibited a substantial accumulation of cells in the S phase (43.8%), indicating accelerated DNA synthesis and active tumor cell proliferation. This was accompanied by a reduction in the apoptotic Sub-G1 fraction (1.3%), suggesting a suppression of programmed cell death within the chemically induced tumors.

The administration of Quercetin (DEN + QR) significantly altered this profile. Quercetin treatment induced a robust accumulation of cells in the G2/M phase (33.7%) and a simultaneous increase in the Sub-G1 population (3.9%). These results indicate that Quercetin disrupts HCC progression by activating pre-mitotic checkpoints and promoting apoptosis. Similarly, exposure to low-dose gamma irradiation (DEN + IR) resulted in a pronounced S-phase arrest (56.2%). This suggests that IR-induced replication stress or DNA damage leads to an accumulation of cells in the synthesis phase, effectively slowing cycle progression.

The most significant findings were observed in the triple combination group (DEN + QR+IR). This regimen induced a profound G2/M arrest (47.2%), significantly higher than that observed in the DEN group or either monotherapy (*p* < 0.01). This enrichment in the G2/M phase occurred alongside a reduction in the S-phase population (10.0%), indicating that the combination therapy effectively halts the transition of cells into and through mitosis.

Furthermore, the Sub-G1 fraction—a marker for DNA fragmentation and apoptosis—reached its highest level in the DEN + QR+IR group (6.0%), demonstrating a superior ability to trigger programmed cell death compared to DEN alone (*p* < 0.001). Combination Efficacy: The DEN + QR+IR group successfully redirected the cell population from active synthesis (S phase) toward a terminal G2/M arrest (47.2%) and increased apoptosis.

### The impact of DEN and monotherapies on annexin V

**Fig. 9:**
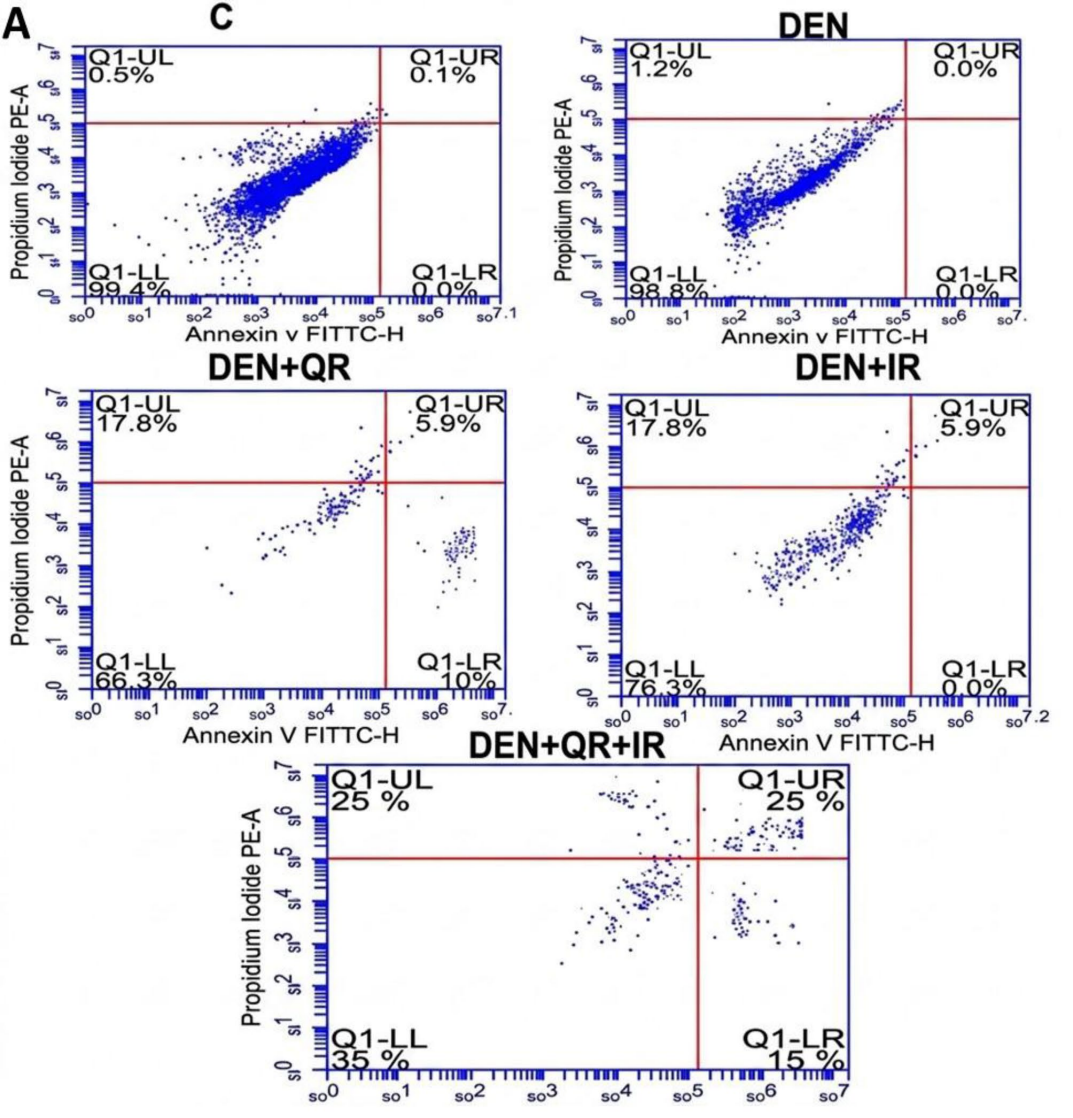

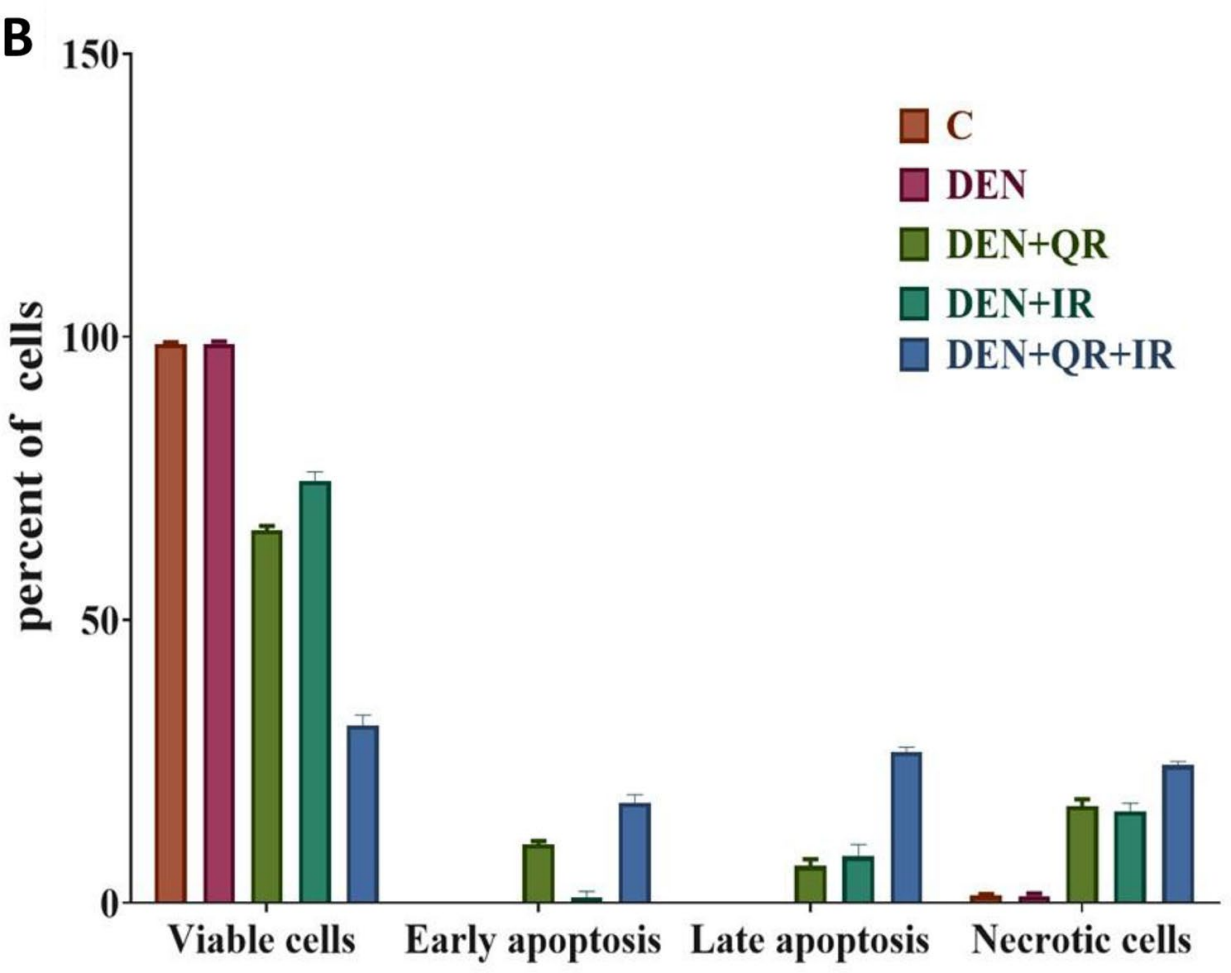
Flow cytometric analysis of apoptosis.Presents the Annexin V/PI dot plots quantifying apoptotic stages in fresh cells. (**A**) Annexin V-FITC/PI Staining Scatter Plots: Representative quadrants illustrating the distribution of cells in various stages of cell death across experimental groups: C (Control), DEN, DEN + QR, DEN + IR, and DEN + QR+IR. The vertical axis represents Propidium Iodide PE-A (PI) staining, and the horizontal axis represents Annexin V FITC-H staining. Quadrants are defined as: Lower Left (Q1-LL): Viable cells (Annexin V−/PI−). Lower Right (Q1-LR): Early apoptotic cells (Annexin V+/PI−). Upper Right (Q1-UR): Late apoptotic cells (Annexin V+/PI+).Upper Left (Q1-UL): Necrotic cells (Annexin V−/PI+). (**B**) Quantitative Apoptosis Data: The bar graph represents the percentage of cells in each stage (Viable, Early Apoptosis, Late Apoptosis, and Necrotic) for the five experimental groups. Data are expressed as the mean ± SEM from independent replicates. Significant differences in cell population distribution are observed between the treated groups compared to the control and DEN groups. Mean % (± SEM, *n* = 3) three independent biological replicates of cell populations

Accordingly, in relation to the divergent apoptotic cascade stages, the way cells attach to annexin V (x axis) and/or PI (y axis) in a flow cytometric dot plot chart aids in differentiating between cells that underwent necrosis (positive PI, negative annexin v staining/upper left quadrant) and cells that underwent apoptosis (annexin V positive cells/lower right quadrant) (Fig. 9). Depending on such strategy, DEN group apoptosis was distinguishably lowered oppose to all the other groups (*p* < 0.05) as presented in Fig. (9). Figure (9A) shows the results of one representative from each group, while Fig. (9B) shows the results of means of three samples from each group. Two distinct populations appeared following the treatment of DEN group with quercitin: one was identified as an early apoptotic cell since it only displayed a single positive annexin V expression, and late apoptotic cells the displayed double positive Annexin V and PI staining (Fig. 9/ upper right quadrant). The early apoptotic cells % increased from 0% in the DEN group to 10% in the DEN groups treated with QR, respectively. It is noteworthy that the highest percentage of apoptotic cells 40% (early and late, the sum of the two quadrant) and the lowest percentage of viable cells (35%) were all reported in the DEN group treated with QR + radiation. In Fig. 9, it is feasible to observe that such populations were concurrent with a gradual and appreciable elevation in the number of necrotic cells, which increased from 1% DEN group to 17.8% in DEN group, treated with QR. moreover irradiation of DEN cells also increase the number of necrotic cells to 18%. Then again, the apoptosis of gamma irradiated DEN treated with QR was significantly higher than their corresponding non- radiated group (*p* < 0.05).

The increase in apoptosis reported for the DEN + QR+IR group was based primarily on the Annexin V/PI assay (Fig. 9), which definitively distinguishes apoptotic from necrotic cells. The sub-G1 peak from PI staining (Fig. 8) was used as supportive evidence of DNA fragmentation and is consistent with the Annexin V data. Collectively, these data suggest that Quercetin acts as a potent radiosensitizer, orchestrating a multifaceted disruption of the cell cycle and enhancing the apoptotic response to low-dose gamma irradiation in HCC.

### Macroscopic examination of the liver tissue

**Fig. 10:**
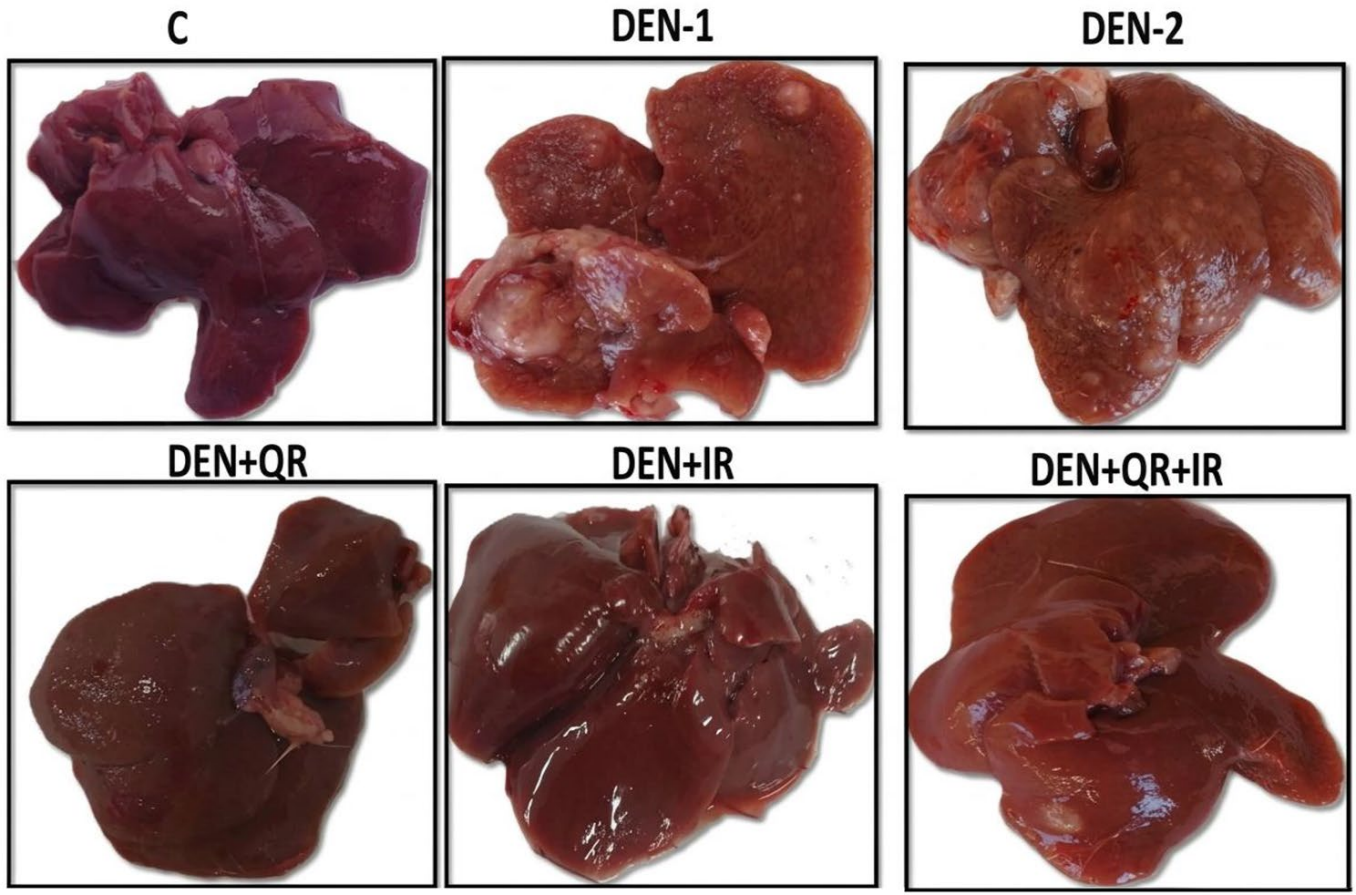
Macroscopic examination of liver tissue across experimental groups. Panels: A = Control, B1, B2 = DEN, C = DEN + quercetin, D = DEN + low-dose radiation (R), E = DEN + quercetin+radiation. The control liver tissue exhibited a smooth, intact surface with normal coloration and well-defined vascular structures (panel A). In contrast, liver tissues from diethylnitrosamine-injected rats showed irregular surfaces with nodules, color changes, and altered textures as shown in (pane B1, B2). Treatment with quercetin resulted in reduced tumor size, and fewer lesions (Panel C). Low-dose radiation exposure demonstrated signs of tissue repair, regeneration, and vascular changes (Panel D). The combined treatment group exhibited enhanced hepatoprotective effects compared to either quercetin or low-dose irradiation alone, with further reduced tumor burden and signs of improved liver function (Panel E)

Across the study groups macroscopic examination (Fig. 10) shows a progression from normal tissue in the control group to increasing tumor burden and morphological changes in the cancer and treatment groups. The effectiveness of interventions using quercetin and/or low-dose radiation is typically assessed by their capacity to restore liver architecture and overall liver health, as illustrated in Fig. 10. In the normal control group, the liver exhibited a smooth, intact surface with normal coloration (pink to light brown) and clearly defined vascular structures (Panel A). Exposure to DEN produced gross liver lesions with irregular surfaces and nodularity (Panel B). Quercetin treatment (Panel C) was associated with fewer lesions and smaller gross tumor areas compared with Panel B. Low-dose radiation (Panel D) appeared to promote tissue repair and vascular remodeling. The combination of radiation and quercetin (Panel E) suggested a trend toward reduced tumor burden and improved architectural normalization relative to the DEN-only group (Panel B).

### Histopathological hepatic alterations

**Fig. 11:**
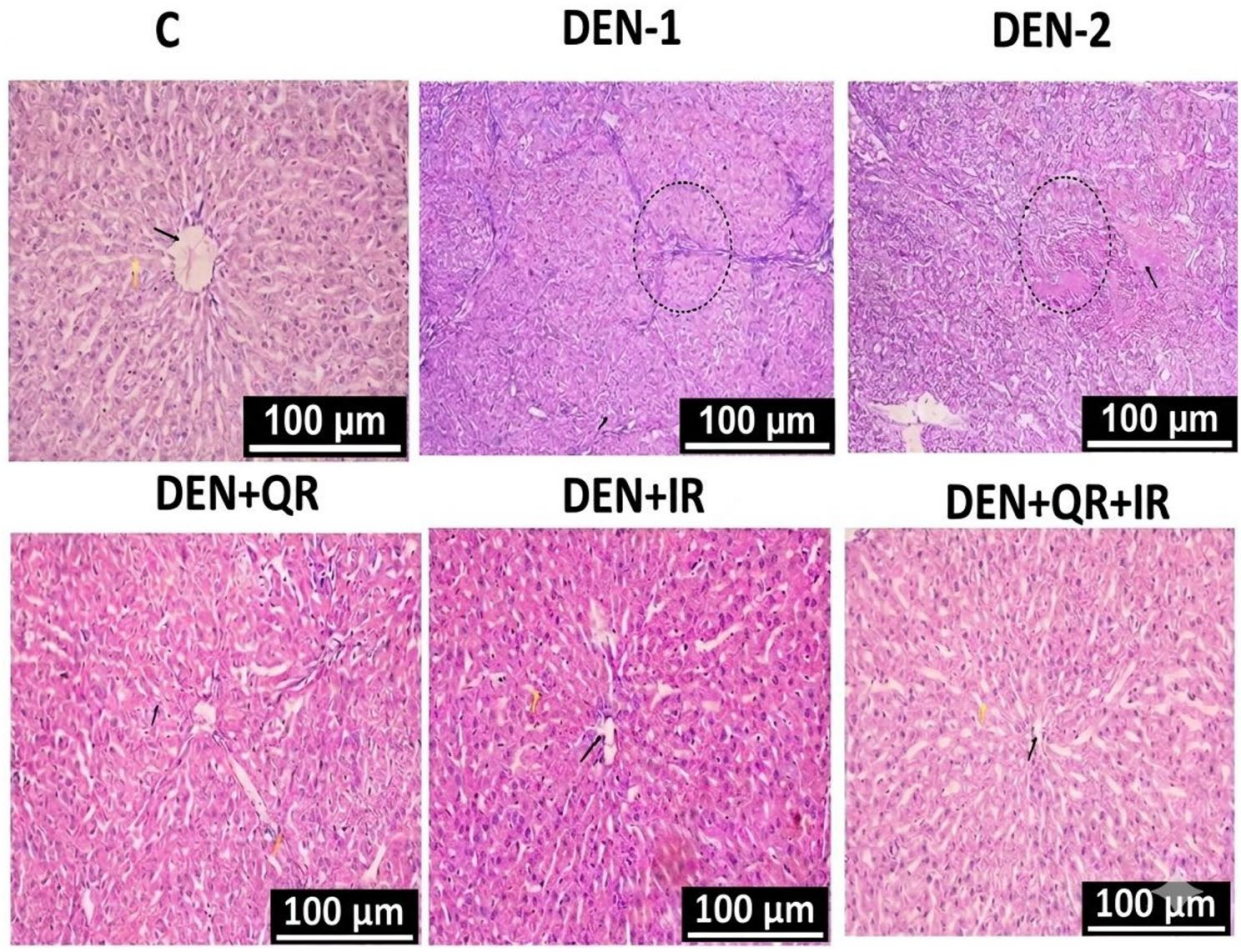
Effect of quercetin and low-dose irradiation on DEN-induced liver histopathology. Representative photomicrographs of H&E-stained liver sections (magnification 100x, scale bar = 100 μm). (**A**) Control: normal hepatic architecture. (**B**) DEN: severe necrosis and fibrosis. (**C**) DEN + IR: partial restoration of hepatic cords. (**D**) DEN + QR: portal inflammation and necrosis. (**E**) DEN + QR+IR: marked improvement with intact central vein. Black arrows: central vein/necrosis; yellow arrows: hepatic cords/inflammation; black circles: fibrotic areas

**Fig. 12:**
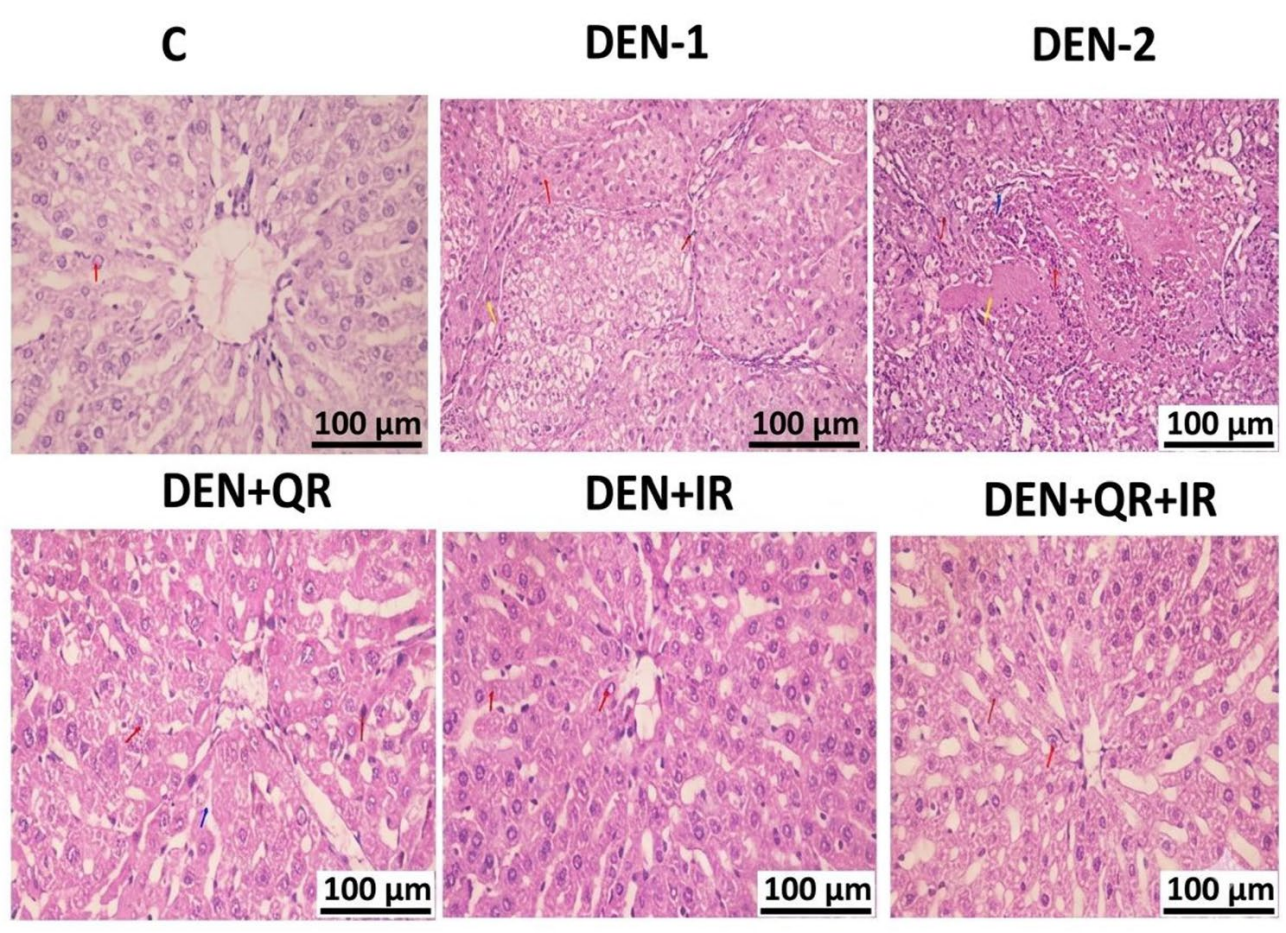
High-magnification histopathology of liver sections showing cellular details. Representative H&E-stained sections (magnification 200x, scale bar = 100 μm). (**A**) Control: normal hepatocytes. (**B**) DEN: extensive degeneration, necrosis, and inflammation. (**C**) DEN + IR: intact hepatocytes with focal necrosis. (**D**) DEN + QR: pyknosis and sinusoidal dilatation. (**E**) DEN + QR+IR: predominantly intact hepatocytes. Red arrows: hepatocytes/mitotic figures; brown arrows: necrosis/pyknosis; yellow arrows: inflammation; blue arrows: sinusoidal changes/fibrosis

Histopathological examination of H&E-stained liver sections revealed distinct morphological alterations across experimental groups (Figs. 11 and 12). The Control group exhibited normal hepatic architecture, with a central vein surrounded by radiating cords of hepatocytes and normal sinusoidal spaces (Figs. 11A and 12A). In contrast, DEN administration induced severe liver injury. At low magnification (100x), DEN-treated livers showed disruption of lobular architecture with extensive bridging fibrosis and pseudolobule formation (Fig. 11B). At higher magnification (200x), foci of well-differentiated hepatocellular carcinoma (HCC) were identified, characterized by cellular atypia, nuclear pleomorphism, increased nuclear-to-cytoplasmic ratio, and abnormal mitotic figures (Fig. 12B). Additional findings included extensive hepatocellular degeneration, coagulative necrosis, marked inflammatory infiltration, and sinusoidal congestion. HCC was confirmed in 4 of 6 animals (66.7%) in the DEN group based on standard histomorphological criteria.

Treatment with quercetin monotherapy (DEN + QR) resulted in only mild histological improvement. Persistent portal inflammation, hepatocyte pyknosis, karyolysis, and sinusoidal dilatation were observed (Figs. 11D and 12D). Residual dysplastic hepatocytes were noted in 1 of 6 animals (16.7%). Low-dose irradiation monotherapy (DEN + IR) showed partial restoration of hepatic cord architecture with residual single-cell necrosis. No definitive HCC foci were observed in this group. Notably, the combination therapy (DEN + QR+IR) demonstrated marked histological improvement, with a predominantly intact central vein, restoration of hepatic cord organization, and only minimal residual changes including mild ballooning degeneration of scattered hepatocytes. No evidence of HCC or severe necrosis was observed in any animal from the DEN + QR+IR group.

**Fig. 13:**
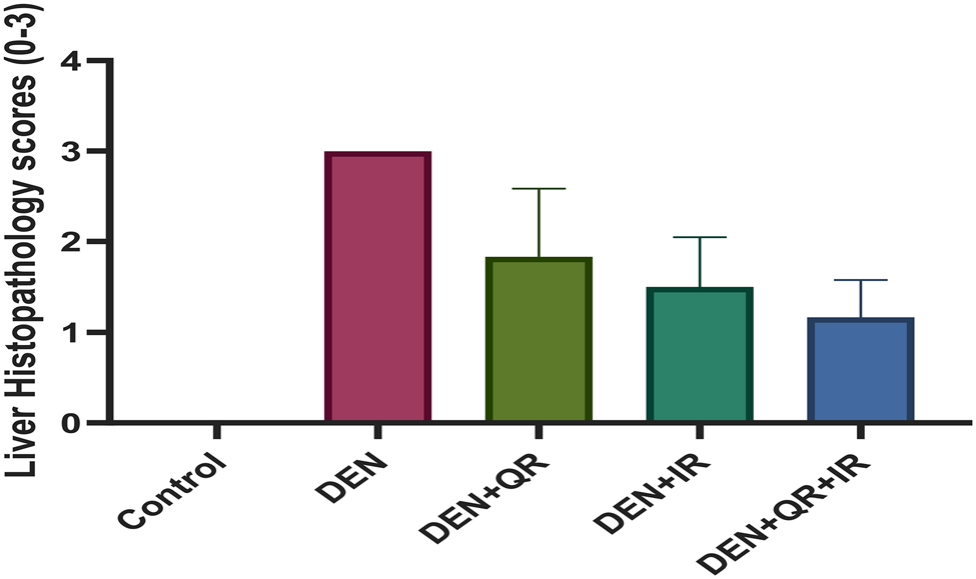
Effect of combination therapy on histopathological liver injury scores. Data are presented as box and whiskers plots showing median, interquartile range, and min-max values. Individual data points represent scores from each animal (*n* = 6/group). Statistical analysis was performed using Kruskal-Wallis test (H = 23.14, *p* < 0.0001) followed by Dunn’s post-hoc test. _p < 0.05, *p < 0.01, *_p < 0.001 vs. Control; #*p* < 0.05, ##*p* < 0.01, ###*p* < 0.001 vs. DEN group

Semiquantitative scoring analysis was performed by a blinded board-certified veterinary pathologist using a 4-point scale (0 = absent, 1 = mild, 2 = moderate, 3 = severe). Data are presented as Median (Range) (Fig. 13) and were analyzed by Kruskal-Wallis test followed by Dunn’s post-hoc test. The DEN group exhibited a median injury score of 3 (range 3–3). Monotherapy reduced the median score to 2 (range 1–3) for DEN + QR and 1.5 (range 1–2) for DEN + IR. The combination therapy produced the greatest reduction, with a median score of 1 (range 1–2). Kruskal-Wallis analysis revealed a highly significant difference among groups (H = 23.14, *p* < 0.0001). Dunn’s test confirmed that all treatments significantly attenuated DEN-induced injury versus the DEN group: DEN + IR (*p* < 0.01), DEN + QR (*p* < 0.05), and DEN + QR+IR (*p* < 0.001). Critically, 5 of 6 animals (83.3%) in the DEN + QR+IR group exhibited only grade 1 (mild) injury, and none showed severe injury (grade 3).

## Discussion

Hepatocellular carcinoma (HCC) accounts for ∼90% of liver cancer cases [31]. In the present study, diethylnitrosamine (DEN) administration recapitulated key hallmarks of hepatotoxicity and early carcinogenesis [32]. DEN increased serum ALT by 9.1-fold and AST by 22.4-fold versus Control (Fig. 1A-B, *p* < 0.001), confirming hepatocellular membrane damage and cytosolic enzyme leakage consistent with DEN-induced injury [21]. This enzyme elevation coincided with a pro-oxidant state: MDA and ROS increased 5.6-fold and 3.6-fold, while SOD and GSH decreased by 76.1% and 77.2% (Figs. 2A-B and 3A-B, *p* < 0.001). Such oxidative imbalance is a known consequence of DEN metabolism by cytochrome P450, generating DNA adducts and lipid peroxidation [33, 34]. Quercetin (QR) treatment attenuated liver injury, reducing ALT by 43.9% and AST by 45.1% versus DEN (Fig. 1A-B, *p* < 0.001). This hepatoprotection was associated with improved redox status: QR decreased MDA by 46.0% and ROS by 36.0%, while increasing SOD by 74.0% and GSH by 73.2% (Figs. 2 and 3, *p* < 0.001). These changes align with the established antioxidant capacity of QR, attributed to its polyphenolic structure that scavenges radicals and chelates metal ions [35, 36]. Similar QR-mediated restoration of SOD and GSH has been reported in MASLD models [37] and hepatocyte cell lines under oxidative stress [38]. Low-dose γ-irradiation (IR) produced comparable effects, reducing ALT by 33.1% and MDA by 38.4% while increasing SOD by 57.0% (Figs. 1, 2 and 3, *p* < 0.001), consistent with adaptive antioxidant responses to low-dose radiation [39, 40]. Importantly, the combination QR + IR produced greater improvements than either agent alone across all injury and redox markers. ALT and AST were reduced by 61.2% and 82.7%, MDA by 70.2%, and SOD and GSH increased by 209.1% and 267.2% versus DEN (Figs. 1, 2 and 3, *p* < 0.001 vs. DEN; *p* < 0.01 vs. monotherapies). This enhanced response suggests that QR and IR act on complementary pathways to mitigate oxidative damage [23].

Inflammation is a key driver of DEN-induced hepatocarcinogenesis [41]. Here, DEN increased NF-κB and STAT-3 by 8.0-fold and 4.4-fold (Fig. 4A-B, *p* < 0.001), along with mTOR and P38 MAPK by 9.0-fold and 10.6-fold (Fig. 5A-B, *p* < 0.001). Elevated NF-κB and STAT-3 sustain hepatic inflammation and tumor-promoting survival signals [42, 43]. QR reduced NF-κB by 44.8% and STAT-3 by 32.8% versus DEN (Fig. 4A-B, *p* < 0.001), consistent with reports that QR interferes with NF-κB and JAK/STAT signaling [36, 44]. IR similarly decreased NF-κB by 34.6% and mTOR by 34.0% (Figs. 4A and 5A, *p* < 0.001). The QR + IR combination achieved the largest suppression: NF-κB -70.2%, STAT-3 -59.0%, mTOR − 64.9%, and P38 MAPK − 78.6% versus DEN (Figs. 4 and 5, *p* < 0.001 vs. DEN; *p* < 0.01 vs. monotherapies) [45, 46]. These data indicate that combined treatment more effectively dampens inflammatory and growth signaling than either intervention alone. DEN also disrupted autophagy and apoptosis balance. Autophagy markers ULK-1, LC3-II, and ATG5 were reduced by 80.0%, 85.5%, and 64.6% (Fig. 6A-C, *p* < 0.001), suggesting compromised autophagic clearance. QR increased ULK-1 by 109.3% and LC3-II by 387.9%, while IR increased them by 50.0% and 333.0% versus DEN (Fig. 6A-B, *p* < 0.001). QR + IR produced the greatest induction: ULK-1 + 318.6%, LC3-II + 531.1%, and ATG5 + 1654.3% versus DEN (Fig. 6A-C, *p* < 0.001 vs. all groups), indicating upregulation of the core autophagic machinery. This aligns with QR-induced protective autophagy via p-STAT3/Bcl-2 [47, 48] and IR-stimulated autophagy for damage clearance [51]. Quercetin sensitizes cancer cells to radiation therapy in vitro and in vivo [49]. Nevertheless, we acknowledge as a limitation of this study that the definitive evaluation of dynamic autophagic flux vs. late-stage flux blockade could not be fully distinguished, owing to the lack of p62 degradation data or dynamic flux inhibitor challenges (such as chloroquine). Future mechanistic investigations will target these dynamic regulatory checkpoints to completely map out the flux kinetics.

Apoptosis resistance was evident with caspase-3 reduced by 74.6% and BCL-2 increased 4.5-fold by DEN (Fig. 7A-B, *p* < 0.001), favoring cell survival [33]. QR reversed this by increasing caspase-3 754.5% and reducing BCL-2 44.0%; IR increased caspase-3 559.1% and reduced BCL-2 29.4% (Fig. 7A-B, *p* < 0.001) [50]. The QR + IR group showed the strongest pro-apoptotic shift: caspase-3 + 1231.8% and BCL-2 -63.2% versus DEN (Fig. 7A-B, *p* < 0.001 vs. all groups). QR-induced apoptosis via STAT3 inhibition and p16-mediated cell cycle arrest has been reported in HCC cells [51, 52]. Quercetin prevents liver carcinogenesis by inducing cell cycle arrest, decreasing cell proliferation and enhancing apoptosis [53]. The enhanced apoptosis with QR + IR correlates with improved histopathology, where the combination group showed the lowest pathology scores and restored liver architecture [54, 55]. QR also impacted cell cycle dynamics. QR alone caused G2/M accumulation and increased sub-G1 populations, indicating mitotic arrest and apoptosis [56]. This is mechanistically supported by QR-mediated downregulation of cyclin B1/CDK1 and induction of p21 via Chk2 (Jeong et al., 2009). IR is reported to induce redox normalization and mitochondrial improvement [57]. Together, QR + IR amplified S-phase arrest and sub-G1 populations, reflecting enhanced anti-proliferative and pro-apoptotic activity.

To contextualize the efficacy of QR + LDR, comparison with current standard-of-care therapies for HCC is relevant. Sorafenib, a multi-kinase inhibitor approved as first-line treatment for advanced HCC, reduces tumor growth and angiogenesis primarily by inhibiting RAF/MEK/ERK and VEGFR/PDGFR signaling [31]. In preclinical DEN-induced HCC models, sorafenib decreases serum ALT and AST by ∼30–45%, reduces NF-κB and STAT-3 expression by ∼40–50%, and induces apoptosis with ∼2–3-fold increases in caspase-3 activity ([43]; Guo et al., 2024). In the present study, QR + LDR combination achieved 61.2% and 82.7% reductions in ALT and AST, 70.2% and 59.0% reductions in NF-κB and STAT-3, and a 13.3-fold increase in caspase-3 versus DEN. While direct head-to-head comparison in the same model was not performed, the magnitude of changes observed with QR + LDR on key injury, inflammation, and apoptosis markers appears comparable to or greater than those reported for sorafenib in similar DEN models. However, sorafenib’s clinical benefit is limited by toxicity and acquired resistance [31]. QR is a dietary flavonoid with a favorable safety profile [36], and LDR at the doses used is below thresholds for overt tissue damage. Therefore, QR + LDR may represent a complementary or alternative strategy with potentially lower toxicity, though clinical studies are required to validate efficacy and safety relative to sorafenib or lenvatinib. Future work should directly compare QR + LDR with standard targeted therapies and immunotherapy in HCC models to define its translational potential.

## Conclusion

This study demonstrates that the combination of quercetin and low-dose gamma irradiation was more effective than either treatment alone in attenuating DEN-induced hepatocellular injury and carcinogenesis in rats. The combination therapy produced the greatest improvements across multiple parameters: it resulted in the largest reductions in serum ALT and AST levels, the lowest median histopathological injury scores, and may prevent the risk of HCC development, whereas HCC was observed in 66.7% of DEN-treated animals. Mechanistically, the combined treatment induced the highest rates of G2/M cell cycle arrest and apoptosis in hepatic tissue, alongside the most pronounced normalization of oxidative stress markers, inflammatory mediators (NF-κB, STAT-3), and growth signaling pathways (mTOR, P38 MAPK), with concomitant restoration of autophagic activity. Independently, quercetin and low-dose irradiation each mitigated DEN-induced hepatic dysfunction, but the combination produced superior outcomes across all endpoints evaluated. These preclinical findings suggest that combining a natural antioxidant with low-dose irradiation may enhance therapeutic efficacy against chemically induced liver injury. However, these results are limited to a single animal model and dosing regimen. Further studies are required to determine the optimal dose and timing, evaluate long-term safety, and validate efficacy in additional models before clinical translation can be considered.

### Study limitations

Despite the significant findings of this research, several limitations should be acknowledged. First, the cell cycle and apoptotic analyses were conducted using single-cell suspensions derived from whole liver tissue. While histopathological examination confirmed a predominance of neoplastic transformation in these samples, the suspension inherently contains a heterogeneous population of cells, including hepatocytes, Kupffer cells, and stellate cells. Consequently, the observed molecular shifts represent the collective response of the liver tumor microenvironment rather than exclusively isolated hepatocellular carcinoma cells. Furthermore, the high mortality observed in the DEN-only group restricted certain biochemical analyses to a sample size of (*n* = 6). However, this mortality reflects the severe, expected hepatotoxicity of the DEN model and was accounted for in our approved experimental protocol, with the validity of our findings further strengthened by the histopathological assessment of all survivors.

Regarding the mechanistic depth of the study, a primary limitation is that autophagic flux was not directly assessed. While we quantified protein levels of ULK-1, LC3-II, and ATG5, we did not determine the LC3-II/I ratio, p62/SQSTM1 degradation, or utilize autophagy inhibitors such as chloroquine to evaluate flux dynamics. Similarly, the specific molecular mechanism of low-dose radiation (LDR) action was not fully explored. While our data are compatible with radiation hormesis, we did not quantify DNA repair proteins, antioxidant response elements (AREs), or heat shock proteins; thus, the proposed hormetic mechanism remains hypothetical. Additionally, while we demonstrated the modulation of Caspase-3 and Bcl-2, the lack of Bax protein data precluded the calculation of the Bax/Bcl-2 ratio. However, our use of Annexin V/PI flow cytometry provides direct, functional evidence of apoptosis at the cellular level to compensate for this.

Finally, certain methodological constraints should be noted. Hepatic ROS levels were assessed using an ELISA kit that measures stable oxidative byproducts rather than the direct detection of transient radicals via DCFH-DA or EPR spectroscopy. While this method is widely utilized in large-scale biochemical studies, direct real-time detection would offer more precise quantification. Nevertheless, the concordant results across multiple oxidative stress markers—including MDA, GSH, and antioxidant enzymes—strongly support our conclusions. Lastly, this study lacked a healthy + LDR control group to directly monitor the effects of 0.25 (x4) Gy on normal liver tissue. However, prior literature has consistently demonstrated that such low doses do not induce significant hepatotoxicity or DNA damage in healthy rodents, supporting the safety profile of the doses utilized in this study.

## Data Availability

No datasets were generated or analysed during the current study.
